# New Insights Into Lignification via Network and Multi-Omics Analyses of Arogenate Dehydratase Knock-Out Mutants in *Arabidopsis thaliana*

**DOI:** 10.3389/fpls.2021.664250

**Published:** 2021-05-25

**Authors:** Kim K. Hixson, Joaquim V. Marques, Jason P. Wendler, Jason E. McDermott, Karl K. Weitz, Therese R. Clauss, Matthew E. Monroe, Ronald J. Moore, Joseph Brown, Mary S. Lipton, Callum J. Bell, Ljiljana Paša-Tolić, Laurence B. Davin, Norman G. Lewis

**Affiliations:** ^1^Institute of Biological Chemistry, Washington State University, Pullman, WA, United States; ^2^Earth and Biological Sciences Directorate, Pacific Northwest National Laboratory, Richland, WA, United States; ^3^National Center for Genome Resources, Santa Fe, NM, United States

**Keywords:** lignin, arogenate dehydratases, multi-omics, network analysis, *Arabidopsis*, ribocode

## Abstract

Multiple *Arabidopsis* arogenate dehydratase (*ADT*) knock-out (KO) mutants, with phenotypes having variable lignin levels (up to *circa* 70% reduction), were studied to investigate how differential reductions in ADTs perturb its overall plant systems biology. Integrated “omics” analyses (metabolome, transcriptome, and proteome) of wild type (WT), single and multiple *ADT* KO lines were conducted. Transcriptome and proteome data were collapsed into gene ortholog (GO) data, with this allowing for enzymatic reaction and metabolome cross-comparisons to uncover dominant or likely metabolic biosynthesis reactions affected. Network analysis of enzymes–highly correlated to stem lignin levels–deduced the involvement of novel putative lignin related proteins or processes. These included those associated with ribosomes, the spliceosome, mRNA transport, aminoacyl tRNA biosynthesis, and phosphorylation. While prior work helped explain lignin biosynthesis regulation at the transcriptional level, our data here provide support for a new hypothesis that there are additional post-transcriptional and translational level processes that need to be considered. These findings are anticipated to lead to development of more accurate depictions of lignin/phenylpropanoid biosynthesis models *in situ*, with new protein targets identified for further biochemical analysis and/or plant bioengineering. Additionally, using KEGG defined functional categorization of proteomics and transcriptomics analyses, we detected significant changes to glucosinolate, α-linolenic acid, nitrogen, carotenoid, aromatic amino acid, phenylpropanoid, and photosynthesis-related metabolic pathways in *ADT* KO mutants. Metabolomics results also revealed that putative carotenoid and galactolipid levels were generally increased in amount, whereas many glucosinolates and phenylpropanoids (including flavonoids and lignans) were decreased in the KO mutants.

## Introduction

**FIGURE 1:**
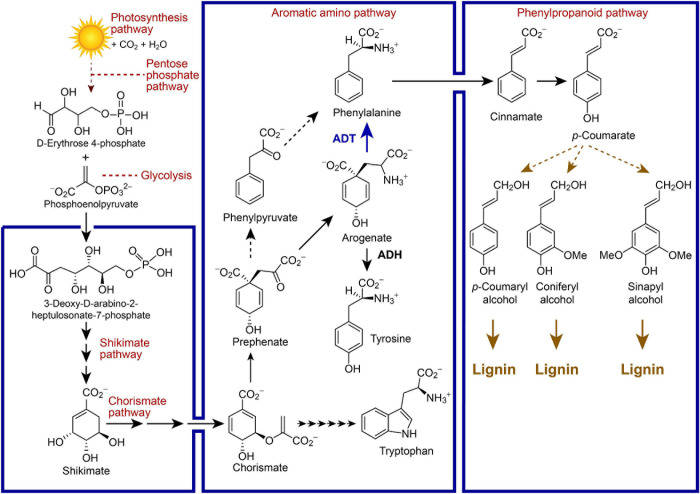
Simplified metabolic pathways linking photosynthesis, carbon fixation and lignin biosynthesis, as well as placement of arogenate dehydratase (ADT) between the chorismate-shikimate, aromatic amino acid, and phenylpropanoid pathways in vascular plant systems. Selected metabolites are illustrated along with enzymes depicted by arrows.

Arogenate is an important branch-point to either tyrosine (Tyr) or phenylalanine (Phe) in vascular plants, whose formation from arogenate (Agn) is catalyzed by arogenate dehydrogenase (ADH) and arogenate dehydratase (ADT), respectively (Figure 1). The Phe so formed can then be incorporated into either proteins or massively utilized in the phenylpropanoid pathway to produce lignins, as well as other related metabolites (e.g., lignans, allyl/propenyl phenols, flavonoids, coumarins, anthocyanins) depending upon the species. In our earlier studies, homozygous single and multiple *Arabidopsis ADT* knock-out (KO) lines with homologous T-DNA insertion mutations were obtained, with the most extreme phenotypes showing an ∼70% reduction in lignin content as compared to wild type (WT) plants (Corea et al., 2012a, b, c). Phenotypes of the former also displayed prostrate stems and lowest stem dry weights/heights.

Differentially varying lignin levels as above, obtained through systematic manipulation of the 6-membered *Arabidopsis ADT* gene family (*ADT1*, At1g11790; *ADT2*, At3g07630; *ADT3*, At2g27820; *ADT4*, At3g44720; *ADT5*, At5g22630, and *ADT6*, At1g08250), thus offered the opportunity to investigate the holistic nature of such perturbations on overall *Arabidopsis* systems biology. To do this, we carried out multi-omics (i.e., transcriptomics, proteomics, and metabolomics) analyses of leaf and stem tissues of single (*adt1*, *adt3*, *adt4*, *adt5*, and *adt6*), double, triple and quadruple (*adt4/5*, *adt1/4/5*, *adt3/4/5*, and *adt3/4/5/6*) KO mutant and WT lines, at 4 weeks of growth/development.

In this study, multi-omics “normalization” strategies were employed to enable direct integration and visualization of the disparate “omics” datasets of related transcripts, proteins and metabolites, thereby allowing for greater understanding, confidence and validation of resulting systemic biological perturbations observed. Network analysis (Szklarczyk et al., 2015) was also used to (a) identify enzymes or processes most highly correlated to different lignin levels, and (b) reveal potentially novel enzymes or processes related with lignin level regulation and biosynthesis. This analysis resulted in our provisionally deducing novel transcriptional, post-transcriptional and translational regulatory processes, when carbon flux was reduced in the phenylpropanoid pathway and which profoundly affected lignin deposition.

Many proteins identified in our network analysis have not been reported as potentially linked to lignin biosynthesis regulation. For example, several ribosome subunits with high correlations to lignin level profiles were identified, suggesting the possible presence of a “ribocode.” These proteins, as well as other ribosome-interacting mRNA processing proteins detected in our network analysis, indicate that vascular plant lignin levels are not only controlled at the point of transcription but work together with modulating factors at a post-transcriptional and translational level as described below.

## Materials and Methods

### Chemicals

For metabolomics analyses, Optima^TM^ grade water, acetonitrile and formic acid were purchased from Fisher Scientific (Hampton, NH, United States). Authentic standards used for secondary metabolite metabolomics are described in Höhner et al. (2018). For proteomics and RNA-Seq sample preparation, nanopure water was used and all chemicals were obtained from Sigma-Aldrich (St. Louis, MO, United States) and were of analytical grade unless otherwise noted.

### *Arabidopsis* T-DNA Knock Out (KO) Insertion

*Arabidopsis thaliana* WT (Columbia) T-DNA insertion lines were obtained from the Salk Institute Genomic Analysis Laboratory (SIGnAL)^1^. Individual *ADT* KO mutant lines were created in homozygous form, with individual lines crossed to ultimately provide double, triple, and quadruple homozygous *ADT* KO mutants (Corea et al., 2012c).

### Plant Growth and Harvesting Conditions

*A. thaliana* WT (Columbia) and KO mutant lines were grown in Institute of Biological Chemistry (Washington State University) greenhouses with a 16/8 h light/dark cycle at 27–28°C/24–26°C, respectively. Nitrogen-based fertilizer (200 ppm) was added five times a week. Plants were harvested at 4 weeks after planting with rosette leaf and stem tissues individually collected and immediately flash-frozen in liquid N_2_. Each sample was stored at −80°C until further analysis.

### Primary Metabolite Derivatization

Nine individual plants of each line were frozen in liquid N_2_ and freeze-dried. Dry plant tissues were ground into fine powders using a tissue-lyzer II (Qiagen, Germany) for 30 s at a frequency of 30 Hz at −80°C. Powdered freeze-dried *Arabidopsis* leaves or stems (20 mg) were individually suspended in 500 μL of methanol:2-iso-propanol:water (5:2:2, v/v/v). After adding ^13^C labeled ribitol standard (1.5 μg), each material was extracted by vortexing for 10 min, followed by sonication for 5 min. Extracts were individually centrifuged for 10 min at 15,000 × *g*, with supernatants transferred to a new vial and subsequently dried under vacuum. Dry residues were individually re-suspended in water:acetonitrile (500 μL, 1:1 v/v), and re-extracted by sequential vortexing and sonication, followed by centrifugation at 15,000 × *g* for 5 min with supernatants then removed and dried under vacuum. Dry residues were individually suspended in *O*-methoxylamine hydrochloride (40 mg mL^–1^ in pyridine, 5 μL, Sigma) and incubated for 90 min at 30°C at 1,000 rpm (Eppendorf Thermomixer). Subsequently, samples were individually derivatized with MSTFA (45 μL) with 1% TMCS (Thermo-Pierce) for 30 min at 37°C and 1,000 rpm (Eppendorf Thermomixer).

### Primary Metabolite Analysis Using Gas Chromatography Time-of-Flight Mass Spectrometry

Analyses were performed using a Pegasus 4D time-of-flight mass spectrometer (LECO) equipped with a Gerstel MPS2 autosampler and an Agilent 7890A oven. Derivatization products were separated on a (30 m × 0.25 mm i.d. ×0.25 μm) Rxi^®^-5Sil column (Restek) with an IntegraGuard^®^ pre-column using ultrapure He at a constant flow of 1 mL min^–1^ as carrier gas. The linear thermal gradient started with 1 min at 50°C, followed by a ramp to 330°C at 20°C min^–1^. Final temperature was held for 5 min prior to returning to initial conditions and mass spectra were collected at 17 spectra s^–1^. The injection port was held at 250°C, and an aliquot of the sample (1 μL) was injected.

### GC-MS Data Processing

Peak annotation employed the Fiehn primary metabolite library (Kind et al., 2009), where an identity score cutoff of 600 was used. Peak alignment and mass spectra comparisons were carried out using the Statistical Compare feature of the ChromaTOF^®^ software (LECO). Surrogate standard ^13^C ribitol and original tissue weight were used for normalization.

### Secondary Metabolomic Analysis Using Liquid Chromatography Time-of-Flight Mass Spectrometry

Metabolites were extracted from freeze-dried material from each individual plant tissue (10 mg) as described (Lu et al., 2017) in 200 μL of methanol:water (70:30, v/v) containing naringenin (Aldrich) as an internal standard. Samples were vortexed, sonicated for 10 min and finally centrifuged at 14,000 × *g* at 4°C for 15 min. Supernatants were individually stored at −80°C until analysis.

Samples were analyzed using a Waters Acquity^TM^ Ultra Performance LC system as described in Lu et al. (2017) with the following modifications: Liquid chromatography was performed using a 100 × 2.1 mm i.d. (1.7 μm particle size) UPLC BEH C18 column (Waters). Masses of eluted compounds were detected using an electrospray ionization (ESI) source in the negative ion mode with capillary voltage at 2.2 kV; cone voltage at 20 eV; collision energy at 6 eV and at 30 eV. Chromatograms and spectra were inspected, and raw data files and metabolite assignments were determined as described previously (Lu et al., 2017). Before statistical analysis, feature integration results were normalized to internal standard naringenin (M271T1197_1, retention time 1197 s and *m/z* (average) 271.0609 and calculated 271.0606, for C_15_H_11_O_5_). Many metabolites were unambiguously identified by comparison with retention times, UV spectra and MS/MS fragmentation patterns of authentic standard compounds. Other metabolites were putatively annotated based on accurate mass, isotopic pattern recognition, and MS/MS spectra. These were searched in both the literature and publicly available mass spectral databases [i.e., Metlin, ReSpect^2^, Massbank^3^, and HMDB^4^ ].

### RNA Extraction and Illumina HiSeq Analysis

For each sample, liquid nitrogen frozen tissue sample (100 mg) was ground using a mortar and pestle cooled in presence of liquid nitrogen. A Spectrum^TM^ Plant Total RNA kit (Sigma-Aldrich, St. Louis, MO, United States) was employed according to the manufacturer’s instructions. Quality control was performed on a Thermo Scientific NanoDrop with all 260/280 nm and 260/230 nm readings above 1.8. Illumina TruSeq RNA libraries were constructed per the manufacturer’s protocol, with quality evaluated using the Agilent Bioanalyzer. Samples were sequenced on a HiSeq 2000 instrument (Illumina, San Diego, CA, United States). Six lanes were multiplexed with five samples per lane in the single ended, 50 bp configuration. A mean of 41 M reads passing quality filters was obtained for each sample (min. 31 M, max. 50 M). Cluster generation used the cBot SR Cluster Gen Kit (V3) and the flow cell used was SR FC Information (V3).

### RNA-Seq Data Workup

CyVerse (formerly iPlant Collaborative)^5^ (Goff et al., 2011), was used for analysis and evaluation of the HiSeq data. Once all FASTQ files produced from the Illumina sequencing were uploaded into the Discovery Environment (DE), the TopHat2-SE App, which comprised TopHat 2.0.9 and Bowtie 2.1.0, was used to align the RNA-Seq reads to the *A. thaliana* TAIR10 genome using the short read aligner Bowtie which is capable of analyzing the mapping results to identify splice junctions between exons. The reference genome and reference annotations used the *A. thaliana* TAIR10 genome (*A. thaliana* Ensembl 19). Default analysis options were used with the exception of the FASTQ quality scale of Illumina 1.3-1.8 (PHRED64). Anchor length: 8; Maximum number of mismatches that can appear in the anchor region of spliced alignment: 0; The minimum intron length: 70; the maximum intron length: 50000; Minimum isoform fraction: 0.15; Maximum number of alignments to be allowed: 20; Minimum intron length that may be found during split-segment (default) search: 50; Maximum intron length that may be found during split-segment (default) search: 500000; Number of mismatches allowed in each segment alignment for reads mapped independently: 2; Minimum length of read segments: 20. Next, Cufflinks2 (an interface for Cufflinks version 2.1.1) located in the DE of CyVerse was used to link short read RNA-Seq alignments found from the TopHat analysis to the reference annotations from the TAIR10 genome. Cufflinks identified sequences that did not align with the annotated genome and labels them with novel identifiers. Default settings were used in Cufflinks. These parameters included abundance estimation options of: Number of importance for samples generated for each locus: 1000; Number of iterations allowed during MLE (maximum-likelihood estimates) of abundances: 5000; Prefix for transcripts in reported GTF (General Transfer Format): Cuff; Minimum isoform fraction: 0.1; Pre-mRNA fraction: 0.1; Maximum intron length: 300000; Alpha value for the binomial test used during false positive spliced alignment filtration: 0.001; Small anchor fraction: 0.09; Minimum fragments per transfrag: 10; The number of bp allowed to enter the intron of a transcript when determining if a read or another transcript is map-able with it: 8; Maximum genomic length allowed for a given bundle: 3500000; Minimum intron length: 50; Minimum average coverage required to attempt 3′ trimming: 10; The fraction of average coverage below which to trim the 3′ end of an assembled transcript: 0.1. Next, the HTSeq-with-BAM-input app in the CyVerse site was used to produce count data from the BAM files using default parameter values.

### Protein Extraction and Digestion

Ice cold 0.1 M ammonium acetate in methanol (10 mL) and β-mercaptoethanol (250 μL) were added to each pulverized powder sample (ground in liquid nitrogen in a mortar and pestle) followed by vigorous shaking for 15 s. For stem tissues, a Polytron homogenizer was used for about 30 s at a speed of 30 to further homogenize them. Samples were individually placed in a −20°C freezer for 2 h, and subsequently centrifuged for 10 min at 5000 × *g* at 4°C. Each supernatant was next removed and discarded. The above addition of ammonium acetate/methanol solution and centrifugation steps were repeated four more times to remove metabolites and lipids from each sample. Excess methanol was removed by drying the pellets gently under a flow of nitrogen for ∼2 min. A protein solubilization solution containing 7 M urea, 2 M thiourea, 4% CHAPS and 5 mM of neutralized tris(2-carboxyethyl)phosphine (TCEP) (Bond-Breaker, Thermo Fisher, San Jose, CA, United States) was added to each sample to completely cover each pellet, plus 500 μL more. Samples were then individually incubated at 4°C overnight. Debris from each was physically mixed into solution with a pipette tip and the slurry sonicated in a Hielscher UTR200 ultrasonic processor for 10–20 s at 100% amplitude. Protein slurries were next individually incubated at 60°C for 30 min, with samples vortexed and sonicated in the sonoreactor again for about 30 s. Each sample was then centrifuged for 10 min at 5000 × *g* at 4°C. A Coomassie Plus protein assay (Pierce, Rockford, IL, United States), using a bovine serum albumin (BSA) standard, was next conducted on individual supernatants to estimate protein contents. Afterward, denatured samples were diluted tenfold with 50 mM ammonium bicarbonate (pH 8.0). CaCl_2_ was added to a concentration of 2 mM and trypsin (Affymetrix, Santa Clara, CA, United States) was added at a trypsin:sample ratio of 1:50 (w/w). Samples were individually digested overnight at 37°C and then alkylated with chloroacetamide at a concentration of 5 mM in the dark at 37°C for 30 min. Peptides from each treatment were desalted with an SCX SPE (SUPELCO Supelclean) using 10 mM ammonium formate (pH 3.0), 25% acetonitrile (v/v) in water, to remove CHAPS, and then a 80:15:5 (v/v/v) methanol:water:ammonium hydroxide (14.8 M) was used to elute peptides. C-18 SPE columns (SUPELCO Discovery) were employed to remove ammonium salts, using a 0.1% TFA (v/v) in nanopure water to wash the peptides and a 80:20 (v/v) acetonitrile:water with 0.1% (v/v) TFA solvent to elute peptides. Peptides were quantified using a BCA assay (Pierce, Rockford, IL, United States) with a BSA standard.

### iTRAQ Peptide Labeling

Peptides were labeled with 8-plex iTRAQ (AB Sciex, Redwood City, CA, United States) reagents: Each peptide sample (30 μg) was placed in a new tube and dried down. Dissolution buffer (iTRAQ buffer kit, 13 μL) was added to each sample, these being vortexed into solution and centrifuged briefly to draw sample to the bottom of each tube. iTRAQ reagent (10 μL) was diluted further with isopropanol (35 μL), and this was added to each sample. Each reaction was carried out at room temperature for 2 h, with 50 mM ammonium bicarbonate (200 μL) added to quench each reaction. After 1 h, contents from all iTRAQ channel reactions were added to one tube and then the sample was vortexed and dried down in a centrifugal vacuum concentrator.

### Offline Fractionation of Peptides and Preparation of Proteome Samples

Labeled peptides were separated using an off-line high pH (pH 10) reversed-phase (RP) separation with a Waters XBridge C18 column (250 mm × 4.6 mm i.d., 5 μm particle size) and a guard column (4.6 mm × 20 mm) using an Agilent 1200 HPLC System. Each sample loaded onto the C18 column was washed for 15 min with Solvent A [10 mM ammonium formate, adjusted to pH 10 with ammonium hydroxide (14.8 M)]. The gradient started with a linear increase of 0% Solvent B [10 mM ammonium formate, pH 10, acetonitrile:water (90:10 v/v)] to: 5% Solvent B over 10 min, 45% Solvent B over 65 min, and then to 100% Solvent B over 15 min. Solvent B was held at 100% for 10 min, and then this was changed to 100% Solvent A, this being held for 20 min to recondition the column. The flow rate was 0.5 mL min^–1^. A total of 48 fractions were collected for each sample into a 96 well plate throughout the above LC gradient. The high pH RP fractions were then combined into 12 fractions using the concatenation strategy previously reported (Wang et al., 2011). Peptide fractions were dried down and re-suspended in nanopure water at a concentration of 75 ng μL^–1^ for mass spectrometry analysis using an LTQ-Orbitrap Velos MS (Thermo Scientific) system as described below.

### Mass-Spectrometry Based Analysis of Peptide Samples

The LC system was custom built using two Agilent 1200 nanoflow pumps and one Agilent 1200 cap pump (Agilent Technologies, Santa Clara, CA, United States), various Valco valves (Valco Instruments Co., Houston, TX, United States), and a PAL autosampler (Leap Technologies, Carrboro, NC, United States). Full automation was made possible by custom software that allows for parallel event coordination and therefore near 100% MS duty cycle through use of two trapping and analytical columns. Reversed-phase columns were prepared in-house by slurry packing 3 μm Jupiter C_18_ (Phenomenex, Torrance, CA, United States) into 40 cm × 360 μm o.d. ×75 μm i.d. fused silica (Polymicro Technologies Inc., Phoenix, AZ, United States) using a 1-cm sol-gel frit for media retention (Maiolica et al., 2005). Trapping columns were prepared similarly by slurry packing 5-μm Jupiter C_18_ into a 4-cm length of 150 μm i.d. fused silica and fritted on both ends. Mobile phases consisted of 0.1% formic acid in water (A) and 0.1% formic acid in acetonitrile (B) operated at 300 nL^–1^ min with a linear gradient profile as follows (min:%B): 0:5, 2:8, 20:12, 75:35, 97:60, 100: 85. Sample injections (5 μL) were trapped and washed on the trapping columns at 3 μL min^–1^ for 20 min prior to alignment with analytical columns. Data acquisition lagged the gradient start and end times by 15 min to account for column dead volume that allowed for the tightest overlap possible in a two-column operation. The two-column operation also allowed for columns to be “washed” (shortened gradients) and re-generated off-line without any cost to duty cycle.

The LTQ Orbitrap Velos mass spectrometer was operated in the data-dependent mode acquiring higher-energy collisional dissociation (HCD) scans (*R* = 7,500, 5 × 104 target ions) after each full MS scan (*R* = 30,000, 3 × 106 target ions) for the top ten most abundant ions within the mass range of 300 to 1,800 *m/z*. An isolation window of 2.5 Thomson units (Th) was used to isolate ions prior to HCD. All HCD scans used normalized collision energy of 45 and maximum injection time of 1000 ms. The dynamic exclusion time was set to 60 s and charge state screening was enabled to reject unassigned and singly charged ions.

### Peptide Identification and Quantification

For peptide identification, MS/MS spectra were searched against a decoy *A. thaliana* protein TAIR10^6^ database using the algorithm SEQUEST (Eng et al., 1994). Search parameters included: no enzyme specificity for proteome data and trypsin enzyme specificity with a maximum of two missed cleaves, ±50 ppm precursor mass tolerance, ±0.05 Da product mass tolerance, and carbamidomethylation of cysteines and iTRAQ labeling of lysines and peptide N-termini as fixed modifications. Allowed variable modifications were oxidation of methionine and proline. MS-GF (Kim et al., 2008) spectra probability values were also calculated for peptides identified from SEQUEST searches. Measured mass accuracy and MS-GF spectra probability were used to filter identified peptides to <0.4% false discovery rate (FDR) at spectrum level and <1% FDR at the peptide level using the decoy approach. iTRAQ reporter ions were extracted using the MASIC software (Monroe et al., 2008) with a 10 ppm mass tolerance for each expected iTRAQ reporter ion as determined from each MS/MS spectrum.

#### Protein and Transcript Relative Fold-change, *Z*-score and *P*-value Determinations

For the protein data, relative abundances of peptides were determined using iTRAQ reporter ion intensity ratios from each MS/MS spectrum. Individual peptide intensity values were determined by dividing the base peak intensity by the relative ratio associated with each reporter ion. Each iTRAQ experiment references a pool of peptides so that all experiments could be linked. All peptide data were combined into a single data table and were transformed to a log_2_ value, following which each data row of each iTRAQ experiment was shifted so that the reference pool value in each iTRAQ experiment was equal. Then each column of data representing each channel of each iTRAQ experiment was mean centered. All peptide values were next transformed to undo the log_2_, by calculating 2 to the power of each data point. Peptide abundance values were then separated into two datasets, one of peptides unique to a single protein and peptides which are shared between two or more proteins. Peptides were rolled up to a protein value by summing the peptides that belong to each protein in each dataset. Where the same protein was calculated in both datasets, only one protein value was selected in a final protein rollup table with the protein value from the unique peptide rollup given preference if identified in all channels. Individual protein roll-up calculations from the unique or shared peptide table were designated in the final protein roll-up table. KEGG Orthology protein family groups (KEGOs) were also determined for both protein and transcript data. Protein KEGOs were calculated by summing all unique peptide values found within a KEGO. Transcript KEGOs were calculated by summing all transcript count data within a like KEGO. All protein, transcript and KEGO values were converted to a log_2_ value. Log_2_ values below 2 in all protein datasets were removed as these primarily represent noise signals which interfere with *z*-score calculations. All replicates were averaged, and MA plots were constructed for each comparison between the average value of each WT and mutant line. All fold change pairs analyzed in MA plots, were combined into a single dataset and the values obtained in the A axis were ranked from lowest to highest. A sliding window which represented 10% of the total number of data point was used to calculate the *z*-score value along the A axis for each data point (in the MA plot). *Z*-score (*z* = x-μ/σ = (datapoint–median)/standard deviation). This standardized the log_2_ fold change distribution among all data points (low and high abundance alike). From the normal distribution of all log_2_ fold changes, a *p*-value was calculated for each log_2_ fold change comparison using the normsdist function in Excel.

### Network Analysis

All proteins with a high Spearman rank correlation (rho > 0.85) to the monomeric guaiacyl (G) and syringyl (S) lignin-derived moieties, and which had a *z*-score ratio value no more than ±0.5 in the *adt1 versus* WT analysis, were identified in WT and *adt1*, *adt3*, *adt4*, *adt5*, *adt4/5*, *adt1/4/5*, *adt3/4/5*, and *adt3/4/5/6* mutants, and were entered into the STRING network analysis algorithm^7^ (Szklarczyk et al., 2017) searching against the *A. thaliana* database. STRING returned protein-protein associations, type of association, and strength of association. Known interactions from either curated databases or which were experimentally determined were considered, along with predicted interactions from gene fusion, gene co-occurrence, gene neighborhood experiments, as well as from text mining, co-expression datasets and protein homology datasets. Node interactions were selected only if STRING returned a minimum interaction score of 0.4 or higher and for nodes with at least one interaction. These data were then exported into an interaction table, this then being made into a SIF document and uploaded into Cytoscape 3.4.0 (Smoot et al., 2011). In Cytoscape, the layout attribute Organic was selected under yFiles Layout. Nodes in close proximity were further clustered together manually based on either like KEGG or a gene ortholog (GO) term functional category if no KEGG category existed. Nodes were represented by rectangles colored by a red-blue color scheme to represent *z*-scores of log_2_ ratios (*ADT* KO mutant/WT), where red represents proteins higher in abundance in *ADT* KO mutants compared to WT, white symbolizes no change, and blue represents proteins higher in abundance in WT compared to *ADT* KO mutants. The thickness of the edge represents strength of relationship between nodes/proteins, as determined by the final combined score output of STRING. A more focused STRING analysis was carried out just on ribosome, spliceosome and mRNA processing involved proteins to view the putative specific connections. Only associations related to known and predicted interactions were considered. A minimum interaction score of 0.4 or higher was used for nodes with at least one interaction.

## Results

A multi-omics evaluation of *Arabidopsis ADT* KO mutant lines (compared to WT plants) was conducted at 4 weeks of growth/development, when the plants had fully developed rosette leaves and had stems about 10–20 cm high (∼1/3 height at maturity). For both tissue types, full RNA-Seq transcriptomics, iTRAQ 8-plex multiplexing proteomics coupled with LC-MS/MS, together with primary (GC-MS) and secondary (LC-MS) metabolomics analyses were performed. Data interrogation resulted in confident detection (1% FDR) of 27,181 transcripts, 8,672 proteins, together with 132 primary and 30 secondary metabolites.

### Normalization of Expression Data for Multi-Omics Integration

Each biomolecular class (i.e., transcripts, proteins, and metabolites) was measured using disparate workflows. Since only a few replicate analyses were feasible, as is typical for RNA-Seq and proteome analysis, variance estimations were offset by “borrowing” information from genes or proteins across the entire analysis (Subramaniam and Hsiao, 2012). The general rationale for this approach relied on the assumption that variance is similar for biomolecules of similar abundances. Transcript or peptide measurements with similar abundances can therefore act as pseudo-replicates producing similar distribution parameters. This strategy is commonly utilized for expression data and with various bioinformatics tools [e.g., SAM (Tusher et al., 2001), limma (Ritchie et al., 2015), and VAMPIRE (Hsiao et al., 2005)].

We applied this approach to log_2_ fold change transcriptome, proteome and metabolome data between each *ADT* KO mutant and WT analysis. Data were first processed as MA-plots (Dudoit et al., 2002; Labbe and Dudoit, 2012) (Supplementary Figure 1), an approach that distributes log_2_ fold change data (*M* = *y*-axis) according to average abundance (*A* = *x*-axis) of the two measurements being compared. Log_2_ fold change calculations for any type of expression data (gene/protein/metabolite) typically form a normal distribution, with subsequent utilization of localized data (or a sliding window of log_2_ fold change data with similar abundances) enabling calculation of more accurate *z*-scores and *p*-values for significance estimations. Resulting *z*-score calculations then allowed for normalization of data into units of standard deviation (away from an average or zero-fold change value). This normalization then permits integration of datasets obtained on disparate platforms (i.e., Illumina sequencing, mass spectrometry) to be compared directly.

### Metabolomics Analysis

Metabolite profiles represent the ultimate molecular phenotypic indicator of an organism at a specific growth/developmental stage. Metabolites often function as cofactors, energy sources, signaling molecules, polymer precursors (e.g., to cellulose, lignins, proteins, DNA), as well as defense, attractant and effector molecules. In the context of plant bioengineering, metabolomic analyses provide the ultimate validation as to which metabolites are increased or decreased in quantity in response to a genetic change or other perturbation. Here we used two mass spectrometric methods to evaluate the differences between each *ADT* KO mutant and WT in terms of both primary and secondary metabolism.

Supplementary Figures 2, 3 show heat maps displaying *z*-scores of log_2_ fold changes between *ADT* KO mutants and WT for the metabolites identified by GC-MS (i.e., primary metabolites) and LC-MS (i.e., secondary metabolites), respectively. Data were grouped by functional categories, as defined by KEGG^8^ (Kanehisa et al., 2017), when available. *Z*-score values pertaining to Supplementary Figures 2, 3 are found in Supplementary Table 1. The GC-MS results (Supplementary Figure 2) are shown as a heat map displaying *z*-score of the log_2_ fold change values of each metabolite found in each *ADT* KO mutant compared to WT. The largest primary metabolite classes identified were related to carbohydrates, amino acids and lipids. Fold-changes found in the carbohydrate class included on average decreases in ascorbate (2.6 in leaf, 2.3 in stem) and dehydroascorbate (2.6 in leaf, 1.5 in stem). While not detected in leaf tissue, melibiose (3.2 fold), and xylitol (2.3 fold) were lower in abundance in the stems.

In both leaf and stem tissues, the most significant change of all metabolites identified from GC-MS data was that of an increase in sorbitol (5.9 fold in leaf, 152 fold in stem). Knocking out even a single *ADT* isoform in *Arabidopsis* has profound effects on sorbitol biosynthesis, and perhaps on a plant-wide level. Jain et al. (2010) reported that treatment of maize seedlings with increased concentrations of sorbitol (a) decreased total chlorophyll, protein and RNA contents, while increasing proline levels and nitrate reductase activity; and (b) induced a stress response that overall inhibited maize growth. By producing so much sorbitol *in vivo*, *ADT* KO mutants may too be experiencing changes in chlorophyll, protein and RNA levels. Moreover, these plant lines may also be under increased levels of stress relative to WT plants. Glycolic acid (2.1 fold in leaf and 2.3 fold in stem) additionally was found to be increased in *ADT* KO mutants *versus* WT. Glycolic acid is produced when ribulose bisphosphate (RuBisCo) fixes O_2_ instead of CO_2_. The increased presence of glycolic acid in both leaf and stem tissues suggests increased photorespiration pathway utilization, which results in a release of CO_2_ back to the atmosphere.

In stem tissue, other carbohydrates such as D-glyceric acid (2.8 fold), 6-deoxy-D-glucose (10.1 fold), and fructose-6-phosphate (4.1 fold) also had higher levels in *ADT* KO lines *versus* WT, whereas significantly increased levels of D-glucoheptose (36.1 fold), allose (4.0 fold), gluconic lactone (3.5 fold), sorbose (2.5 fold), threonic acid (7.3 fold), and glucose-1-phosphate (3.9 fold) were observed in leaves.

Amino acids with the most increased levels in the *ADT* KO mutants *versus* WT stem tissues included allothreonine (4.2 fold), alanine (2.3 fold), and glutamate (2.2 fold). By contrast, decreases in the *ADT* KO lines were found for oxoproline (2.4 fold) in the stems. Interestingly, shikimate which is a key metabolite located in the aromatic amino acid/shikimate chorismate pathways upstream of ADT, was, on average, increased (2.6 fold) in *ADT* KO mutant leaves, but decreased (8.3 fold) in abundance in stem tissues.

The LC-MS metabolomics analysis of hydro-alcoholic extracts of leaves and stems led to the detection of numerous important secondary metabolites (Supplementary Figure 3). This approach was chosen based on previous knowledge of important and characteristic metabolites produced by *A. thaliana*, which include flavonoids, phenylpropanoids (e.g., sinapate esters, lignans) and glucosinolates. We found that putative galactolipids and carotenoids generally increased in abundance in both tissues, whereas most of the identified flavonoids, 1-*O*-β-*D*-glucopyranosyl sinapate, 5-hydroxyferuloyl malate and lignans (e.g., putative dimeric coniferyl-OH hexoside), generally decreased in abundance most prominently in the stem tissues, with the greatest reductions being in stems with single or multiple KOs of *ADT5*. Nine glucosinolates, which are nitrogen- and sulfur-containing metabolites synthesized by plants as defense against herbivory and pathogens, were also identified. Glucosinolates derived from homomethione (i.e., glucoiberin, glucohirsutin, 7-methylsulfinylheptylglucosinolate, and glucohesperalin) generally decreased in abundance in the *ADT* KO mutants compared to WT, whereas those derived from Trp or Phe showed mixed abundance changes depending on the *ADT* KO mutant and tissue type. Glucobrassicin, 4-hydroxyglucobrassicin and 1-methoxyglucobrassicin, all derived from Trp, showed increases and decreases depending on the specific *ADT* KO mutant analyzed. 4-Methoxyglucobrassicin (Trp derived) increased in abundance in most *ADT* KO mutants leaf samples, and in most of the double/triple/quadruple *ADT* KO mutants as well as in *adt5*. Ties between glucosinolates and phenylpropanoids, with lignin being synthesized in the phenylpropanoid pathway, have been previously documented in the literature, suggesting that there is a strong link between these two pathways. For example, Kim et al. (2015) identified an indole glucosinolate which limits phenylpropanoid accumulation via an inhibitory effect on early steps of phenylpropanoid biosynthesis.

### Transcriptomics and Proteomics Analysis

Various metabolites can often be synthesized or metabolized by one or more isoenzymes contained in distinct plant compartments and/or expressed at different times within a plant’s lifespan. This makes metabolite identification and quantification alone not entirely sufficient to convey key information on specific gene/enzyme targets necessary for full understanding of a system or for bioengineering purposes. For this reason, transcriptomics and proteomics prove to be invaluable for identifying significantly affected pathways, specific gene/enzyme targets, gene regulation events, and tissue specific and/or subcellular compartment-specific target enzyme information.

Supplementary Figure 4 shows RNA-Seq counts observed in leaves and stems for each line investigated. In WT plants, the relative order of transcript abundance in stems (where lignin deposition is greatest) was determined to be *ADT5* > *ADT4* > *ADT3* > *ADT1* > *ADT6*. Earlier phylogenetic evaluation showed that ADT3, ADT4 and ADT5 are all part of the same phylogenetic subgroup. It was also determined that these three ADTs correspond to proteins that exclusively utilize arogenate as a substrate (Cho et al., 2007), whereas ADT1, ADT2, and ADT6 showed a preference for arogenate but could potentially, albeit less efficiently, use prephenate in synthesizing Phe. Our findings that *ADT3*, *ADT4*, and *ADT5* were highest in abundance in 4 week old stems was corroborated by similar findings of RT-PCR levels found in 5 week old *Arabidopsis* stem tissues (Corea et al., 2012c) and separate Northern blot analyses (Rippert et al., 2009) in 4 week old WT *Arabidopsis*. The latter showed that *ADT4* and *ADT5* were highest in abundance in stem and root tissues.

RNA-Seq data also indicated an increase in *ADT* expression between *ADT3*, *ADT4*, and *ADT5*, when one of the *ADT*s in that subgroup was knocked-out. Large increases of *ADT5* were observed in the stems when *ADT3* and *ADT4* were knocked out, showing ∼2 fold and ∼4 fold increases in the *adt3* and *adt4* knockout lines, respectively. When *ADT5* was knocked out, *ADT4* was expressed in greater amounts (∼2 fold increase). Overall, these data showed that, especially in stem tissue, *ADT* mRNA expression between *ADT3*, *ADT4*, and *ADT5* seemed to be coordinated, as remaining *ADTs* in the *ADT3/ADT4/ADT5* subgroup increased when other subgroup members were knocked out. In leaf tissue, only modest effects were observed within this subgroup.

To view coverage and relative amounts (i.e., counts) of mRNA fragments identified in each RNA-Seq analysis (in reference to each *ADT* in leaves or stems), location of the various T-DNA insertions used to produce the KOs and peptides identified from the proteomics analysis, gene product data for each gene were illustrated side by side. The IGV Browser (Robinson et al., 2011; Thorvaldsdóttir et al., 2013) was used to map out reads to the gene, and using a PNNL developed script^9^, the peptide data were converted into BED files which could then be used by any genome viewer software. This allowed visualization of proteomics data in relation to the gene alongside the RNA-Seq data.

Supplementary Figure 5 displays an example of gene-transcript-peptide visualization comparisons detected in WT and *ADT* KO lines for *ADT5*. Comparisons of all other *ADT*s in all RNA-Seq and proteomics data can be accessed at the MassIVE and ProteomeXchange data repository sites (Accession numbers MSV000081518 and PXD007701, respectively). Read sequences downstream of transcription from T-DNA insertions displayed vastly decreased levels as expected. The proteomics data also showed that few peptides from ADTs were identifiable in the high throughput analysis here, which indicated that the ADTs were likely low in abundance relative to the entire proteome in the leaves. By contrast, in stem tissues, more ADT transcripts and peptides were generally identifiable, supporting the hypothesis that *ADT* genes, as well as proteins, were relatively higher in abundance in stem tissues compared to leaves. This is likely due to stem tissues needing to biosynthesize lignin in greater relative amounts compared to leaves.

We next examined what proportion of each KEGG functional category, on average, contained the most significantly (*p*-value < 0.05) changed proteins and transcripts in both leaf and stem tissues in all *ADT* KO lines (Supplementary Figure 6). Categories with the greatest proportion of significantly changed proteins/transcripts were involved in glucosinolate biosynthesis, α-linolenic acid metabolism, nitrogen, carotenoid and phenylpropanoid biosynthesis, as well as aromatic amino acid metabolism. These findings are in high agreement with the significant changes observed in the metabolomics data (Supplementary Figures 2, 3).

Protein and transcript analyses of the putative carotenoid biosynthesis pathway showed a high degree of significantly changed (*p*-value < 0.05) protein/transcript members (Supplementary Figure 6) when *ADT* content was reduced. Metabolite measurements of putative carotenoids also showed an almost universal increase in abundance in all *ADT* KO lines in both leaves and stems (Supplementary Figure 3). Carotenoids function in plants as accessory pigments in plastid membranes and in chloroplasts they are thought to contribute to energy dissipation and can act as protective agents against reactive oxygen species (ROS) (Nisar et al., 2015). Carotenoids and their cleavage apocarotenoid products, which may also serve as signaling molecules, are important in assembly of photosynthesis and antenna proteins for photosynthesis and photoprotection (Cazzonelli and Pogson, 2010). Protein analysis of photosynthesis and antenna protein biosynthesis pathways showed a relatively high proportion of significantly changed proteins (*p*-value < 0.05) (Supplementary Figure 6A) in *ADT* KO mutants suggesting that knocking out *ADTs* in plants also affects photosynthesis.

While increases in the proportion of significantly changed proteins were observed in photosynthesis and carbon fixation pathways, especially in stems, such large changes were not seen at the corresponding transcript level. The fact that mRNA pools related to photosynthesis and carbon fixation were not changed much, whereas translation copy numbers were largely increased, raises the possibility of a post-transcriptional regulatory mechanism. Other categories which were markedly different between protein and transcript data included those involved in Phe metabolism, and glycolate/decarboxylate metabolism. These were found to be highly changed in the protein data, especially in stem tissues, but less so at the transcript level. Categories that showed relatively high changes at the transcript level, but did not seem to translate to higher changes at the protein level, included those pathways involved in alanine/aspartate/glutamate, propanoate, and galactose metabolism, respectively. This was not surprising given that ADTs biosynthesize Phe.

We also observed that shikimate-chorismate derived metabolism pathways (i.e., to Phe, Tyr, and tryptophan) were highly changed at both transcript and protein levels. It is known that Phe, Tyr, and Trp produced via the shikimate-chorismate pathway contribute to feedback inhibition mechanisms of various shikimate pathway enzymes upstream of ADT (Tzin and Galili, 2010), and that *ADT* KOs can alter not only Phe levels but also levels of Tyr and Trp (Corea et al., 2012a). We thus hypothesize that in *ADT* KO mutants there likely may be alterations in the levels of other metabolites synthesized from Phe/Tyr/Trp precursors. These could potentially affect metabolites including phytohormones, which could, in turn, produce system-wide effects. For instance, phytohormone indole-3-acetic acid (IAA) is synthesized from Trp, and IAA can affect changes in growth, development and metabolism (Zhao, 2010).

Another phytohormone potentially altered in *ADT* KO mutants may be jasmonic acid (JA). While we did not specifically detect JA or any other phytohormone in the untargeted metabolomics data, changes in JA precursors may be indicative of potential changes in the downstream phytohormone levels. JA is derived from α-linolenic acid (Weber, 2002) and α-linolenic acid metabolism pathways were highly changed at both the transcript and protein level (Supplementary Figure 6). α-Linolenic acid is not only a precursor to JA, but also aids in photosynthetic thylakoid membrane fluidity (Yashroy, 1987) potentially acting as another contributor to alterations affecting photosynthesis. Other studies indicated that JA levels have a strong relationship to lignin levels. Denness et al. (2011) showed that both JA-isoleucine production and ROS production could form a negative feedback loop which can then repress each other’s production and influence lignin accumulation. Given there were lower levels of ascorbate and dehydroascorbate levels in the metabolomes derived from both leaf and stem tissue of *ADT* KO mutants compared with WT, it may be that ROS is higher in concentration in *ADT* KO mutants given that there is less ascorbate to scavenge ROS. This along with altered levels of JA could potentially be contributing signals affecting lignin biosynthesis. Ascorbate is also a cofactor for biosynthesis of several other phytohormones, such as ethylene, gibberellins, and abscisic acid (Pastori et al., 2003). Due to the potential for all these various phytohormones to be altered in amounts when *ADT* is knocked out, we hypothesize this may be why we see multiple pathway enzymes and metabolite levels affected and not just those directly upstream or downstream of *ADT*.

### Integrated Metabolome, Proteome, and Transcriptome Analysis

**FIGURE 2:**
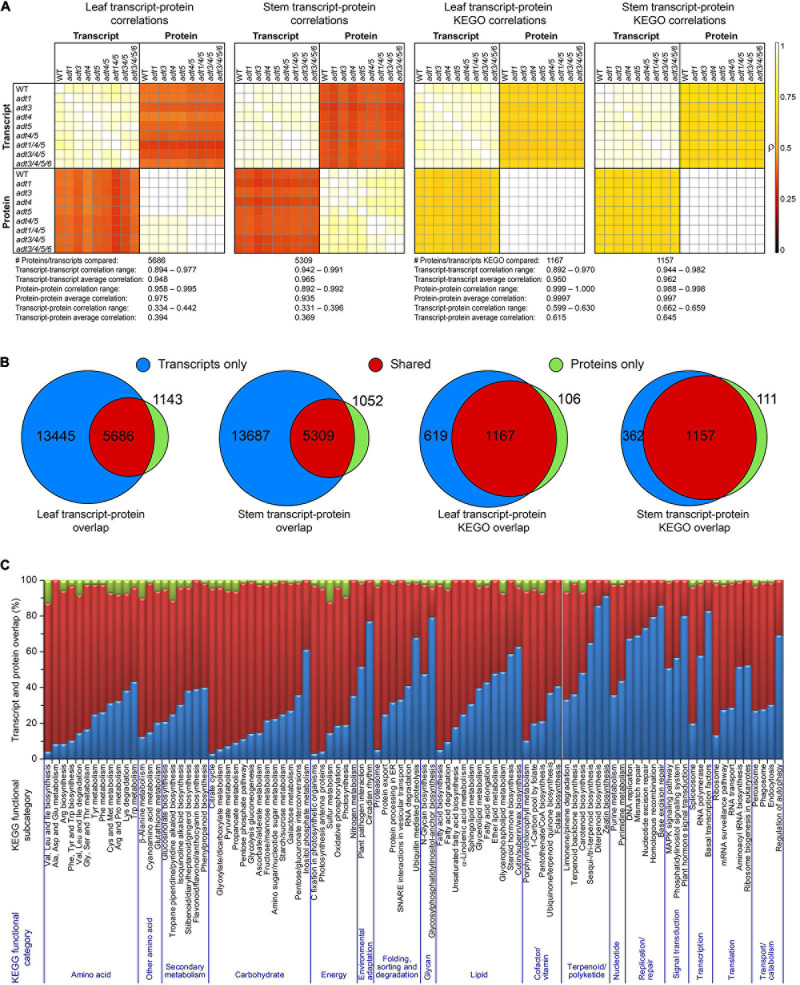
Proposed correlations of transcript and protein data. **(A)** Pearson pairwise correlation plots of each log_2_ abundance ratio data between each *ADT* KO mutant versus WT dataset compared with all others in both the transcript and protein data. The two leftmost plots are from transcript and protein data, and the last two plots represent collapsed transcript and protein KEGG ortholog data (KEGO) for leaf or stem sample sets. **(B)** Venn diagram transcripts and proteins, and transcripts and protein KEGOs identified in leaf and stem data. **(C)** Transcript and protein overlap in all protein and transcript data according to KEGG functional category.

Figure 2A shows Pearson’s Pairwise log_2_ abundance dataset correlations between each WT and *ADT* KO mutant, as compared to every other *ADT* KO mutant between both proteins and transcripts at the gene product (protein or transcript) and KEGG ortholog (KEGO) enzyme family levels. Transcripts and proteins had an average correlation of 0.394 and 0.369 in leaf and stem data, respectively. Correlations increased, however, when data were further collapsed into KEGO comparisons, with abundance correlations on average being 0.615 and 0.645. Only about 30 and 28% of the transcriptome was detected at the protein level in leaf and stem data (Figure 2B), respectively, whereas KEGO level coverage was about 65%. About 17% of proteins had no detectable transcript, and about 8% of protein KEGOs had no corresponding detectable transcript KEGOs. Overlap percentages between detectable transcripts and proteins also varied due to the KEGG functional category of which they were a member (Figure 2C). Proteins present which no longer have a detectable transcript might represent long-lasting proteins or protein degradation products remaining in the cells after degradation of the corresponding mRNA have occurred.

**FIGURE 3:**
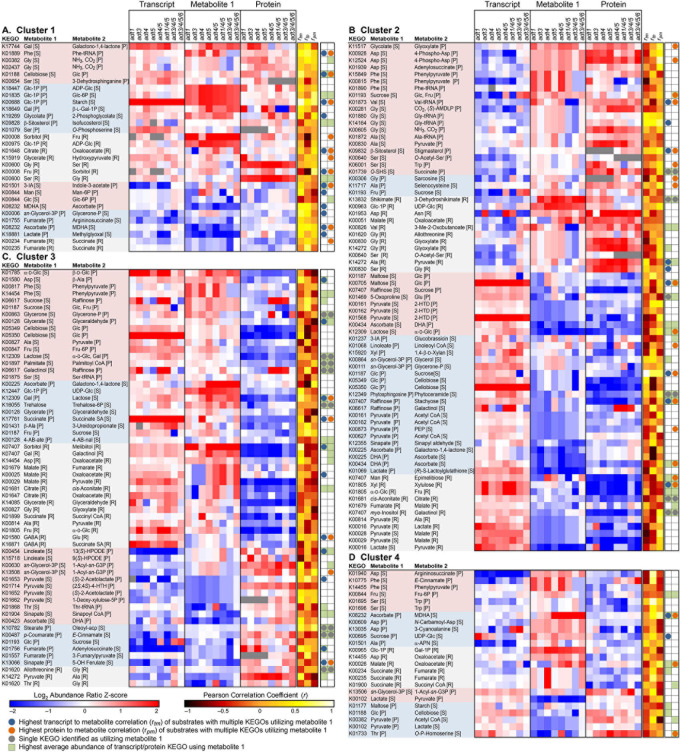
Transcript-metabolite-protein heatmap displaying *z*-score comparisons of the log_2_ ratio pairs (*ADT* KO mutant/wild type, WT) of identified metabolites found to have a corresponding KEGG Ortholog Gene Family (KEGO) member which utilizes that specific metabolite as a substrate, and which were detected in both transcript and proteomics data. Each KEGO is displayed with substrate and product metabolite formed by that KEGO enzyme family. Data is further sorted into clusters that show: **(A) Cluster 1**–Transcripts, metabolites and proteins which all on average increased or decreased in the *ADT* KO mutants together compared to WT. **(B) Cluster 2**–Transcripts decreased, while metabolites and proteins in the *ADT* KO lines, on average, increased in abundance. **(C) Cluster 3**–Transcripts and metabolites on average in the *ADT* KO lines increased, and proteins decreased in abundance compared to WT. **(D) Cluster 4**–Transcripts and proteins decreased, and metabolites on average increased in the *ADT* KO mutants compared to WT. In each cluster, entries are further grouped by whether or not Metabolite 1 is a substrate or product in a unidirectional reaction or if it can be utilized in a reversible reaction. Reactions are then ordered from highest average metabolite *z*-score to lowest metabolite *z*-score. Red represents metabolites higher in abundance in the *ADT* KO mutant compared to WT, blue represents metabolites higher in abundance in WT compared to the *ADT* KO mutant, white represents metabolites unchanged in abundance between WT and the *ADT* KO mutant, and grey represents constituents not detected. Green squares indicate the highest average KEGO value associated with each detected metabolite. Grey circles represent KEGO reactions where there was only a single known reaction for that given substrate-product reaction. Blue circles represent log_2_ transcript data that is most highly correlated to log_2_ metabolite data across *ADT* KO mutants, i.e. if ratio abundances between transcripts and metabolites both showed profile increases across single, double, triple and quadruple *ADT* KO mutants, those would have a positive correlation regardless if the *z*-score values themselves were negative or positive. Orange circles represent log_2_ protein data that are most highly correlated to log_2_ metabolite data across *ADT* KO mutants, i.e. if ratio abundances between proteins and metabolites both showed profile increases across single, double, triple and quadruple *ADT* KO mutants, those would have a positive correlation regardless if the *z*-score values themselves were negative or positive. Abbreviations: *r*_*tm*_ = Pearson’s Correlation between transcript and metabolite profiles. *r*_*pm*_ = Pearson’s Correlation between protein and metabolite profiles. *r*_*tp*_ = Pearson’s Correlation between transcript and protein profiles.1-Acyl-*sn*-G3P, 1-Acyl-*sn*-glycerol 3-phosphate; 2-HTD, 2-(α-Hydroxyethyl)thiamine diphosphate; 3-IA, 3-Indole acetonitrile; 9(*S*)-HPODE, 9(*S*)-Hydroperoxy octadecadienoic acid; 13(*S*)-HPODE, 13(*S*)-Hydroperoxy-(9*Z*,11*E*)-octadecadienoic acid; (2*S*,4*S*)-4-HTH,(2*S*,4*S*)-4-Hydroxy-2,3,4,5-tetrahydrodipicolinate; α-APN, aAminopropiononitrile; DHA, Dehydroascorbate; GABA, 4-Aminobutanoate; MDHA, Monodehydroascorbate; 4-AB-ate, 4-Acetamidobutanoate; 4-AB-nal, 4-Acetamidobutanal; Oleoyl-acp, Oleoyl-[acyl carrier protein]; *O*-SHS, *O*-Succinylhomoserine;(*S*)-AMDLP, (*S*)-Aminomethyldihydrolipoylprotein; Succinate SA, Succinate semialdehyde.

To integrate transcript, protein and metabolomics data for a more direct comparison, KEGO values were compared. In this way, since protein and transcript values reflect an overall summed contribution to each enzymatic reactions, their comparisons are likely closer to metabolite abundance changes which themselves are tissue-wide summed values. *Z*-scores of each log_2_ abundance ratio transcript-metabolite-protein reaction set are displayed in Figure 3 (leaf data) and Supplementary Figures 7, 8 (stem data), respectively, with corresponding data in Supplementary Tables 2, 3. As shown in the transcript-metabolite-protein heatmaps, there are multiple enzyme KEGOs that can utilize the same substrate, but which could produce different metabolite products. Moreover, sometimes there are multiple KEGOs which carry out the same reaction.

We further clustered reactions into four groups: Cluster 1 (Figure 3A, and Supplementary Figures 7A, 8A) represent reactions which on average all increased or decreased in abundance in *ADT* KO mutants compared to WT samples for transcript, protein and metabolite data; Cluster 2 (Figure 3B and Supplementary Figures 7B, 8B) depict KEGO reactions where metabolites and protein KEGOs either both increased or decreased together in abundance in *ADT* KO lines compared to WT, and where transcripts had an opposite abundance change to metabolites; Cluster 3 (Figure 3C and Supplementary Figures 7C, 8C) are of reactions where transcripts and metabolites either both increased or decreased together in abundance in *ADT* KO lines as compared to WT, and where proteins showed the opposite abundance change; and Cluster 4 (Figure 3D and Supplementary Figures 7D, 8D) are of KEGO reactions where protein and transcript abundance changes moved in an opposite direction to metabolite abundance changes between *ADT* KO mutant and WT lines.

Alongside *z*-score values for each KEGO and metabolite, indicators were added to show the reaction that represented the most abundant KEGO for multiple KEGO reactions for a given metabolite. Indicators were also added for which KEGO abundances were most highly correlated to metabolite abundances. Additionally, KEGO reactions were further grouped into categories of whether or not the detected metabolite was either substrate or product in each reaction, or if it could be involved in a reversible reaction (i.e., a metabolite that can serve as both substrate and product).

**FIGURE 4:**
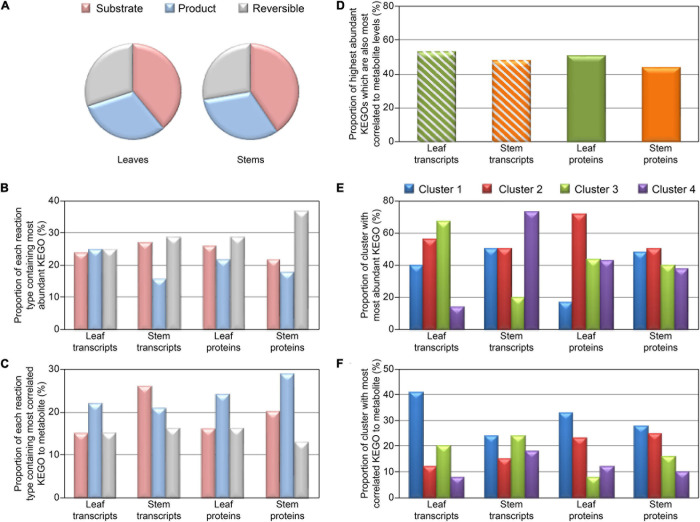
Distribution plots for KEGO reaction heat maps displayed in Figure 3, and Supplementary Figures 7, 8. Distribution of KEGO-metabolite reactions for detected metabolites which are substrates or products in unidirectional reactions or reversible reactions **(A)**. **(B)** Proportion of each KEGO-metabolite reaction type containing the most abundant KEGO. **(C)** Proportion of each reaction type containing the most correlated KEGO to metabolite, when there are multiple KEGOs which react with a metabolite. **(D)** Proportion of highest abundant KEGOs which also are most correlated to metabolite level. **(E)** Proportion of each cluster (defined in Figure 3 and Supplementary Figure 7) which contain the most abundant KEGOs. **(F)** Proportion of each cluster which contained the highest correlated KEGOs to metabolite, when there are multiple KEGOs which react to metabolite level.

Figure 4 shows distribution of reaction types (unidirectional substrate to product, or reversible) and proportion of each reaction type with KEGOs most correlated to metabolite levels, and KEGOs of greatest abundance (when there are multiple KEGOs for a given metabolite). Interestingly, for the protein KEGO data, where a given reaction type showed highest proportion of the most abundant KEGOs, it then had the least proportion of highest correlations between protein and metabolite abundances (Figures 4B,C). When comparing protein KEGO and metabolite abundance changes, we also observed that the most highly abundant KEGOs were not usually the most correlated with detected metabolite level (Figure 4), i.e., most abundant enzymes did not necessarily produce the most metabolite. Given that metabolite abundances are determined by multiple factors not represented by enzyme abundance alone (i.e., enzyme kinetics, differential degradation rates, and post-translational de/activation of enzymes), this observation is not surprising. Indeed, we can generalize that only ∼45–50% (Figure 4D) of the most abundant KEGOs produced metabolite levels that were most highly correlated to KEGO abundances. Metabolite levels controlled by other KEGOs, with low correlations to metabolite levels, may also be influenced by enzyme kinetics and post-translational activation/deactivation. Figure 4E showed that in Cluster 2 (i.e., transcripts are negatively correlated to proteins and metabolites) of the transcript-metabolite-protein comparisons, the most abundant KEGOs were present in both the leaves and stems. In contrast, Figure 4F had a negative correlation in both the leaves and stems, and in both transcript and protein data. Cluster 1 (i.e., transcript, proteins and metabolites are all positively correlated), and had the highest correlated KEGOs profiles to metabolite profiles.

### Network Analysis

Lignin levels were measured previously in the *ADT* KO mutant and WT lines by Corea et al. (2012c) A Spearman’s rank correlation between the *ADT* KO mutant and WT log_2_ ratios to these lignin levels in 4 week old stems was thus calculated here, with proteins identified with a non-parametric measure of rank correlation (rho) of at least ±0.85. Proteins identified as having a high positive or negative correlation to varying lignin levels across *ADT* KO mutants were entered into the on-line network analysis tool STRING (see text footnote 7) (Szklarczyk et al., 2017). Protein-protein interactions and associations identified from STRING were then used as input data into the network software Cytoscape (Smoot et al., 2011).

*Z*-score data representing abundance changes between *ADT* KO mutants and WT were overlaid on proteins (nodes), with line thicknesses (edges) of connecting proteins representing potential protein association strength as calculated by STRING analysis. Proteins found to be proximal in the initial Cytoscape network analyses were further manually grouped into KEGG functional categories to highlight relationships between functional categories. Supplementary Figure 9B shows a stem network analysis showing the *z*-score changes of multiple *ADT* KO mutant versus WT analyses. As more *ADT*s (and specifically as *ADT3*, *ADT4*, and *ADT5*) were knocked out and lignin levels decreased (Supplementary Figure 9A), protein abundances deviated further away from WT levels as indicated by nodes appearing darker red or blue in color (Supplementary Figure 9B).

**FIGURE 5:**
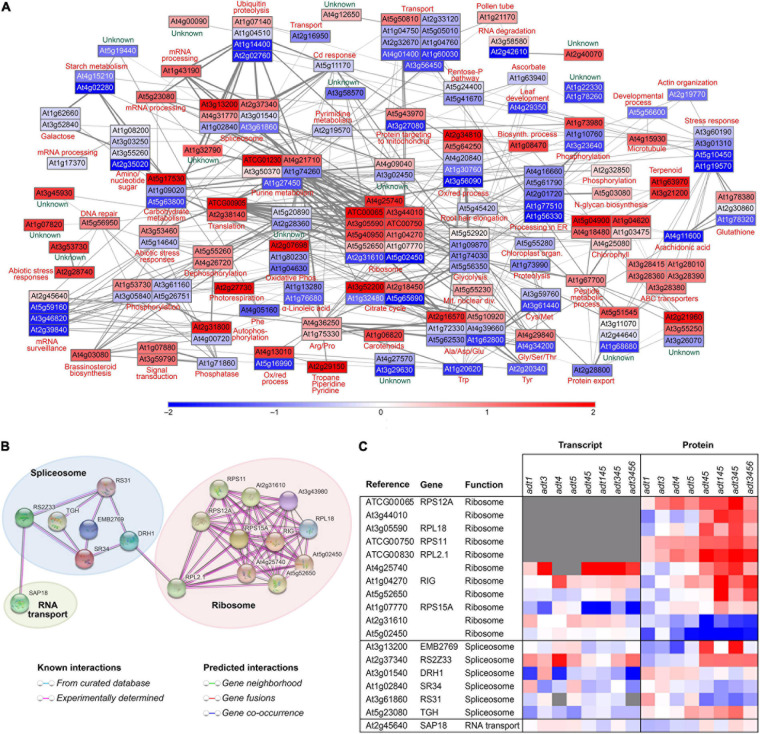
Network analysis and related correlations. **(A)** Network analysis of relative protein abundances determined to be highly correlated in a Spearman rank correlation analysis (rho > 0.85) to lignin cleaved guaiacyl (G) + syringyl (S) monomer levels in 4 week old stem tissues of *adt3/4/5*. Nodes are represented by rectangles colored by the *z*-score of the log_2_ ratios (*ADT* KO mutant/WT), where red represents proteins higher in abundance in the mutant compared to WT, blue represents proteins higher in abundance in WT compared to the *ADT* KO mutant, and white represents proteins unchanged in abundance between WT and the *ADT* KO mutant. **(B)** STRING analysis with solely highly correlated proteins identified in KEGG functional categories associated with ribosomes, spliceosome, and RNA transport showing the direct known and predicted interactions. **(C)** Heatmap showing log_2_ ratio distribution (*ADT* KO mutant/WT) for proteins associated with the ribosome, spliceosome and RNA transport for each plant line for transcript and protein data.

Figure 5A specifically shows the network analysis of *adt3/4/5* stem data as compared to WT, with the *ADT* KO mutant having greatest reduction in lignin (cleavable monomeric G + S) levels as measured in 4 week old *Arabidopsis* stems (Supplementary Figure 9A)–although the lignin level difference between *adt3/4/5* and *adt3/4/5/6* was not statistically different. Enzymes associated with aromatic amino acid, photosynthesis, carotenoid, and α-linolenic acid metabolism were found to be apparently correlated with reduced lignin levels, and this corroborated well with KEGG functional categories identified as being significantly changed (Supplementary Figure 6A). As our focus was in identifying potential lignin-associated proteins in post-transcriptional regulation, known and predicted interactions between mRNA processing and translation machinery proteins were highlighted (see Figure 5B). Figure 5C displays the heat map of corresponding log_2_ *z*-scores of transcript and protein data scrutinized from the mRNA processing and translation machinery for all 8 *ADT* KO lines (Figure 5B).

## Discussion

To begin to put the analyses and discussion below in needed context, it is well known that transcripts and proteins are linked biologically by the central dogma which purports unidirectional flow of genetic information from DNA to RNA to proteins (Crick, 1958). Indeed, comparisons between proteomic and transcriptomic data can highlight which proteins and transcripts have a co-regulatory response (i.e., a high transcript level with a high protein level). The central dogma though is extended in this study to include metabolomics, as many metabolites represent the final product(s) biosynthesized by specific cells/tissues under various environmental conditions or through stresses at specific times during the lifespan of an organism.

### Protein and Transcript Comparisons

In this study, it was found that transcript and protein data between *ADT* KO mutants correlated well within their own groups (i.e., transcript to transcript or protein to protein comparisons). However, correlations on average were quite low for transcript to protein comparisons, being better but still relatively low for transcript to protein KEGO/gene family comparisons. Venn diagrams which depict the overlap between proteins and transcripts revealed that (a) most transcripts detected did not correspond to a detectable protein, likely due to dynamic range and sampling limits of the proteomics technology compared to the depth of coverage capable with RNA-Seq and (b) not all proteins detected corresponded to a detectable transcript (Figure 2) as could arise from differential mRNA and protein degradation rates. Lack of high correlations though between transcripts and proteins have been found to be typical. In many multi-omics evaluations, across eukaryotic and prokaryotic systems, transcripts and proteins are only modestly correlated (Foss et al., 2007; Ghazalpour et al., 2011; Battle et al., 2015; Chick et al., 2016; Hasin et al., 2017). Two examples from plant and animal organisms highlight this point. In a multi-omics investigation of maize, Walley et al. (2016) observed that transcriptome and proteome abundance profiles showed little overlap. In a comparable study of human disease, Hasin et al. (2017) found that transcript levels often exhibited a poor correlation with protein levels, although it was considered that the proteomics data alone was more proximal to the disease mechanisms.

Furthermore, metabolite levels did not show a dominant correlation with either transcripts or proteins. These data illustrate the limitations in using only a single omics evaluation to make systems level biological assessments. Comparisons between these omics datasets may thus reveal (a) what transcripts are actually translated and in what copy number, and (b) what metabolomics data help reveal which proteins or protein families are more or less active in producing the observed metabolites. The integrated “omics” analysis, as presented here, provides over-arching and molecule specific information, which is useful for understanding general molecular pathway trends, and for identifying targets of interest for bioengineering purposes or for follow-on analyses.

### Network Analysis Reveals Lignin/Phenylpropanoid Biosynthesis Associated Proteins

Network analysis is a powerful tool for understanding systems biology effects, drawing from known and predicted direct (physical) and indirect (functional) interactions from multiple data and literature sources. Such analyses can utilize enzyme relationships derived from hundreds of sources (e.g., yeast two hybrid, co-localization, co-expression, literature text mining, etc.). In turn, this can help provide new insights into interconnections and significant relationships of proteins with known and as-of-yet unknown function. Since our main focus was on proteins potentially related to altered lignin levels (as determined by thioacidolysis analysis), we selected those with greatest positive and negative correlative trends to lignin amounts present in *Arabidopsis* stems of each line.

Centrally located in the lignin correlated protein network data created (Figures 5A,B) were eleven ribosomal subunits that correlated with reduced lignin levels. This raised the possibility that lignin levels may be in part regulated by a ribosome filter or “ribocode” (Mauro and Edelman, 2002; Xue and Barna, 2012). That is, lignin biosynthesis regulation may be associated with presence of specific ribosomal subunits involved in translation of lignin-biosynthesis-associated mRNA.

Recent studies have documented evidence for ribocodes, the concept of which suggests differential, temporal and spatial presence of specific ribosomal subunits which preferentially translate specific mRNAs, thus representing regulation at translation. Other observations of possible plant ribocodes include phosphate and Fe-deficiency studies by Rodríguez-Celma et al. (2013) and Wang et al. (2013), who both showed evidence for remodeled translational machinery in response to environmental signals in *Arabidopsis*. Liu et al. (2012) also reported that translational control was mRNA abundance independent, concluding that mRNA levels had less effect on gene activity than translational control mechanisms in *Arabidopsis*.

Other potential targets of lignin biosynthesis regulation identified included those associated with purine metabolism, the spliceosome, RNA degradation, mRNA surveillance and general mRNA processing proteins. Post-translational modifications, protein transport, and targeting mechanisms also appeared correlated with varying lignin levels. Additionally, post-translational related protein processing involving the endoplasmic reticulum was identified, as well as ubiquitin proteolysis, phosphorylation, autophosphorylation, and phosphatase proteins which may be connected to important processes related to lignin biosynthesis.

In our analysis as to what connects the spliceosome, which edits native mRNA into mature translatable mRNA and the ribosomal subunits, was the spliceosome protein DRH1 (At3g01540) to the large ribosomal subunit RPL2.1 (ATCG00830) (Figure 5B). DRH1 is a ATP/dATP-dependent RNA helicase and polynucleotide-dependent ATPase (Okanami et al., 1998). Export of poly(A)(+) RNA has been shown to be greatly blocked in DRH1 mutants (Du et al., 2016). Presence or absence of this helicase could thus serve as a level of regulation, blocking or allowing export of mRNA into the cytosol.

Connected to the identified spliceosome proteins was SAP18 (Song and Galbraith, 2006). In plants, this functions as a transcriptional repressor and associates with ethylene-responsive element binding factors to create a hormone-sensitive multimeric repressor complex under conditions of stress. A SAP18 loss of function mutant produces a plant both more susceptible to salt and impaired in chlorophyll synthesis.

These data indicate what we generally observed with our multi-omics data, namely that: (a) despite any significant presence of mRNA, there are additional factors which may prevent proper processing of mRNA into mature translatable form; (b) there are also other factors which may prevent any mRNA present from ever leaving the nucleus to be translated.

Together, these data also highlight the importance of proteomics to illuminate what protein products are actually present and which are potentially functional.

Furthermore, the vast majority of lignin biosynthesis regulation research thus far has focused on genes and transcriptional control (Nakano et al., 2015). From this body of knowledge, we know that lignin biosynthesis is influenced by a series of transcription factors, such as NAC master switches, which can activate or repress an array of other downstream transcription factors (e.g., MYBs). What is lesser known are what additional post-transcriptional controls of lignin or other phenylpropanoids exist. Due to our discovery that modulating ADTs produces differential lignin contents in *Arabidopsis*, the opportunity was presented where–through this multi-omics study–we could investigate molecular profiles with step-wise decreases or increases in lockstep to varying lignin levels. These results of our investigation suggest that there are potentially multiple processes, phytohormones and layers of regulation involved in lignin biosynthesis or in response to decreased carbon flux through the phenylpropanoid pathway.

Since this study was focused on correlations specific to lignin deposition, most attention was paid to correlative levels found in stems, although other phenylpropanoid-derived biomolecules and other pathways were also affected by *ADT* composition changes in leaves. It may be an interesting and informative exercise to further explore correlative enzyme abundances to other phenylpropanoids that may play a more central role in leaf tissue function. For example, those associated with UV protection (e.g., flavonoids and sinapate esters) (Stapleton and Walbot, 1994; Landry et al., 1995; Mazza et al., 2000; Clé et al., 2008; Sullivan et al., 2014), in order to tease out specific post-transcriptional biosynthesis regulation of formation of these other phenylpropanoids.

Ultimately, the multi-omics investigation and network analysis presented here proved invaluable for understanding systems level changes that modulated ADTs evoke in a model vascular plant. Importantly, it highlighted potentially new levels of post-transcriptional regulation of lignin/phenylpropanoids that can provisionally serve as biomolecular targets for follow-on analyses and/or for bioengineering purposes, i.e., aimed at modulating or modeling lignin/phenylpropanoids in vascular plants. These proteins can thus be used in follow-on lignin-associated enzyme validation studies and/or can potentially serve as truly novel bioengineering targets for manipulation of lignin/phenylpropanoid levels in vascular plants.

### Potential of a Lignin Regulating “Ribocode”

Ribosomes are the effectors in the final steps of gene expression, and it has emerged recently that ribosomes themselves could contribute to regulation via differential ribosomal subunit abundance. Evidence recently has also shown that according to developmental, environmental and pathological conditions, cells can produce different populations of ribosomes which differ in their ribosomal protein and RNA composition. These “specialized ribosomes” suggest that the unique ribosomal composition determines the translational activity of the ribosome and thus controls the biosynthesis of specific proteins and enzymes. For many years, ribosomes were thought to consist of a set number of ribosomal proteins (RPs) and rRNAs, with each RP present as a single copy with resulting conserved stoichiometry and homogeneity. One of the first indications that different types of ribosomes existed was from the study of RP paralogs in plants. In work done by Williams and Sussex (1995), it was found that RPL16 paralog gene expression patterns in *Arabidopsis* were mutually exclusively expressed in different organs of the plant. In yeast studies, deletion of specific RP paralogs also gave rise to unique phenotypes (Ni and Snyder, 2001; Enyenihi and Saunders, 2003). In human studies, evidence came from patients displaying genetic diseases (i.e., ribosomopathies) caused by haploinsufficiency of genes encoding key factors in ribosome biogenesis or RPs (Draptchinskaia et al., 1999). Proteomic-driven analyses exploring composition of purified ribosomes under various conditions identified all RPs differentially expressed between conditions in murine embryonic stem cells, suggesting that different translational statuses were associated with differential stoichiometry among RPs (Slavov et al., 2015).

In our study, we used the STRING (see text footnote 7) algorithm to identify connections/associations between lignin profile correlated proteins. Our results returned interactions between nucleus and plastid proteins (e.g., RPL2.1 and DRH1). While there is currently no direct evidence in *Arabidopsis* that this association exists, there is growing documentation of interactions between homologs of these proteins in other organisms (e.g., humans, yeast, *Escherichia coli* and *Helicobacter pylori*) (Garcia-Gomez et al., 2011; Havugimana et al., 2012). Additional studies, however, would need to be performed to verify the actual association, if any, between eukaryotic and plastid translation-associated proteins in *Arabidopsis*.

These studies indicating the influence of differential ribosomal subunit stoichiometry on differential phenotypes and/or translational products suggest that the ribosome has the ability to function in a regulatory fashion in translation. These supporting data, along with observations of correlative associations between specific ribosomes and lignin production in this study here, support the hypothesis that lignin levels in vascular plants may be partially controlled by specialized ribosome compositions or a lignin “ribocode.”

## Data Availability Statement

The datasets presented in this study can be found in online repositories. The names of the repository/repositories and accession number(s) are: https://massive.ucsd.edu/ProteoSAFe/static/massive.jsp, MSV000081518; proteom ecentral.proteomexchange.org/cgi/GetDataset, PXD007701.

## Author Contributions

KH, CB, ML, LP-T, LD, and NL: conceptualization. KH and JMa: formal analysis. NL, LD, and KH: funding acquisition. KH, JMa, JW, JEM, KW, TC, MM, RM, JB, LD, and NL: investigation and methodology. NL, KH, LD, ML, and LP-T: project administration. KH and LD: visualization. KH: writing–original draft. KH, CB, LD, and NL: writing–review and editing. All authors have read and agreed to the published version of the manuscript.

## Conflict of Interest

The authors declare that the research was conducted in the absence of any commercial or financial relationships that could be construed as a potential conflict of interest.

## Footnotes

1 http://signal.salk.edu/cgi-bin/tdnaexpress

2 http://spectra.psc.riken.jp/

3 http://www.massbank.jp/

4 http://www.hmdb.ca/

5 http://www.cyverse.org/

6 www.arabidopsis.org

7 https://string-db.org

8 http://www.kegg.jp/

9 https://gist.github.com/brwnj/de6007a9a4c652d05028e381be4c7207
