# Supplementary material for: New Insights Into Lignification via Network and Multi-Omics Analyses of Arogenate Dehydratase Knock-Out Mutants in Arabidopsis thaliana
Source: Front Plant Sci. 2021 May 25;12:664250. doi: 10.3389/fpls.2021.664250 (PMC8185232; doi:10.3389/fpls.2021.664250)
Supplement: Supplementary file 1 [file Data_Sheet_1.zip › Supplementary Figures.pdf]

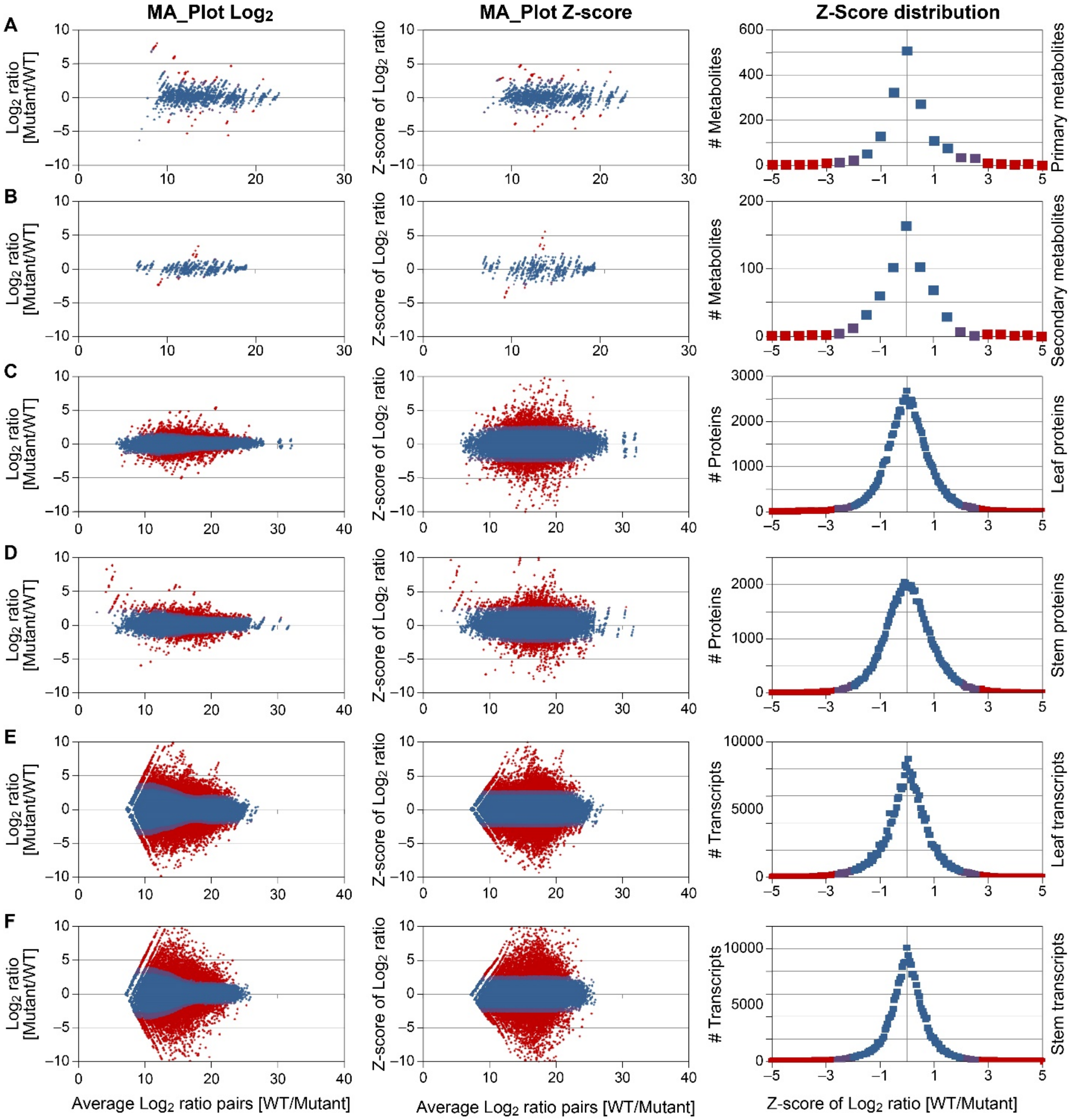

Supplementary Figure 1. MA plots depicting either the $\log_2$ ratios (leftmost plot) or the z-score of the $\log_2$ ratio (middle plot) as determined from the z-score histogram distributions (rightmost plot) of **(A)** GC-MS (primary) and **(B)** LC-MS (secondary) metabolomics data, **(C)** leaf proteomics data, **(D)** stem proteomics data, **(E)** leaf transcriptomics data, and **(F)** stem transcriptomics data. Data points all represent a ratio of paired data (*ADT* KO mutants/ wild type, WT). Blue data points have a p-value $\geq$ 0.05, purple data have a p-value $\geq$ 0.01 and <0.05, and red data points have a p-value <0.01.

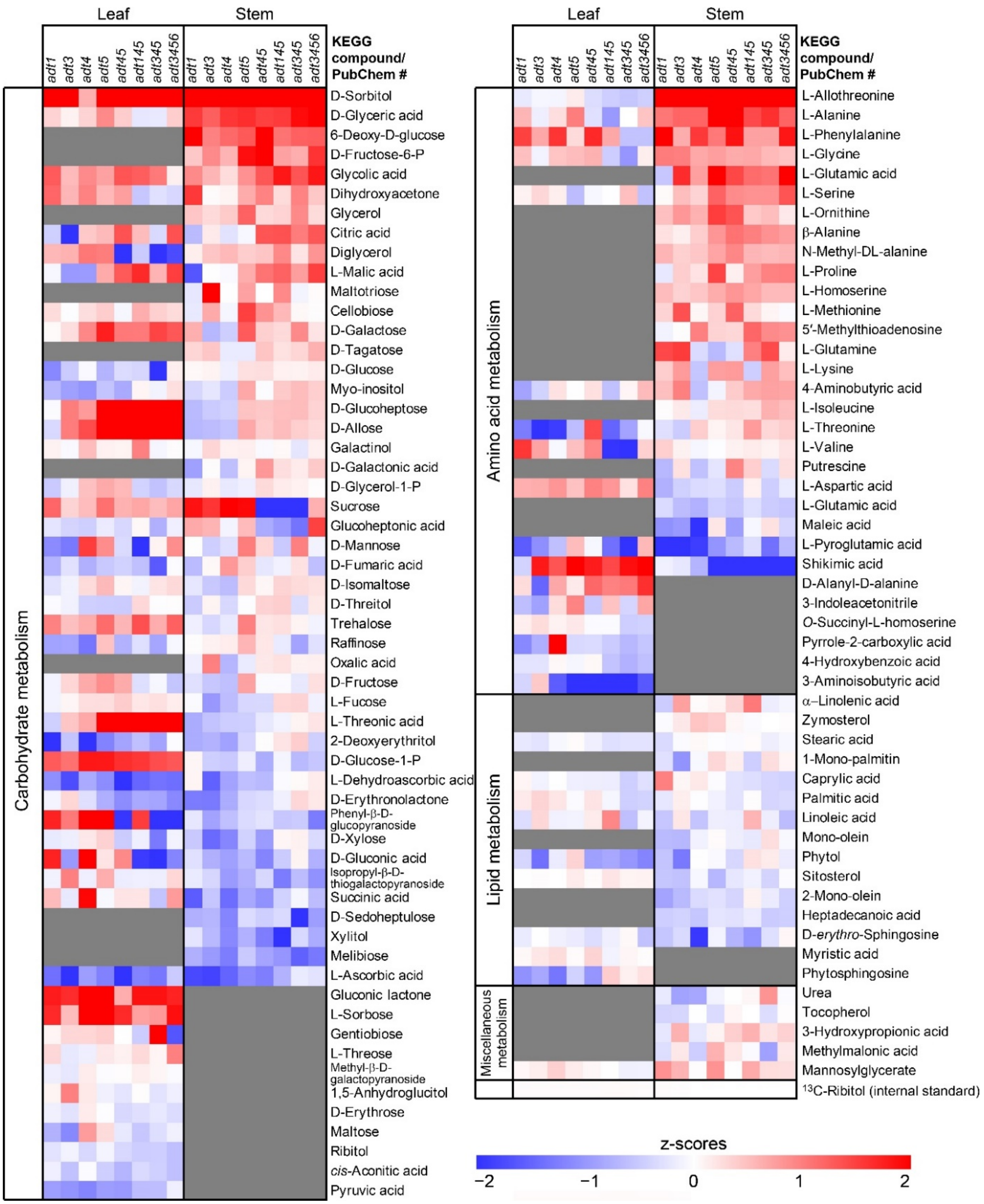

Supplementary Figure 2. Heatmaps showing the z-score value of each metabolite $\log_2$ ratio pair (*ADT* KO mutant/wild type, WT) in the GC-MS primary metabolite analysis. Metabolite data were clustered into compound classes and/or KEGG functional category. Red represents metabolites higher in abundance in the *ADT* KO mutant compared to WT, blue represents metabolites higher in abundance in WT compared to the *ADT* KO mutant and white represents metabolites unchanged in abundance between *ADT* KO mutant and WT. Grey represents undetected constituents. Identification based on Kind et al. (2009).

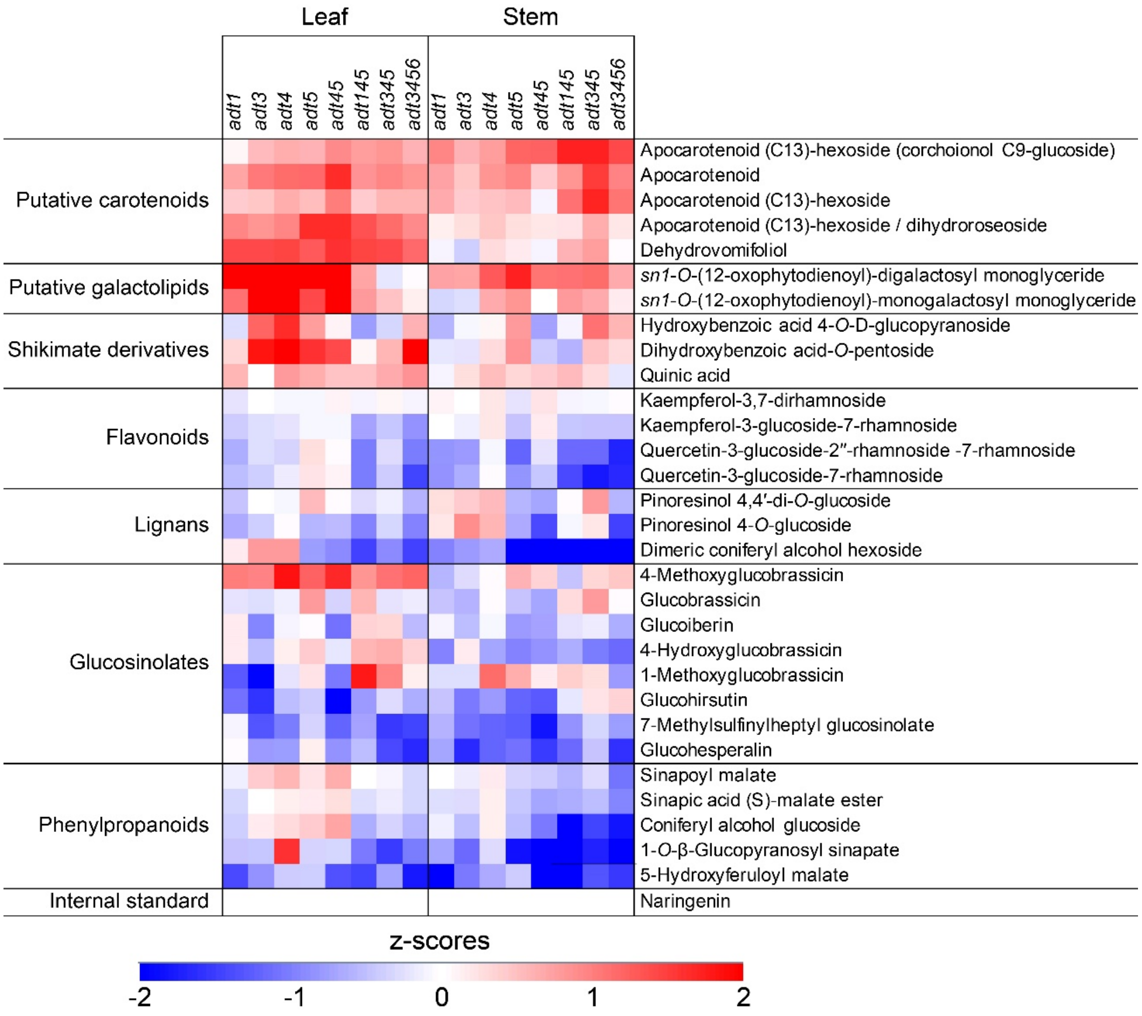

Supplementary Figure 3. Heatmap showing the z-score value of each metabolite $\log_2$ ratio pair (*ADT* KO mutant/wild type, WT) in the LC-MS secondary metabolite analysis. Metabolite data were clustered into general compound classes. Red represents metabolites higher in abundance in the *ADT* KO mutant compared to WT, blue represents metabolites higher in abundance in WT compared to the *ADT* KO mutant, and white represents metabolites unchanged in abundance between WT and the *ADT* KO mutant.

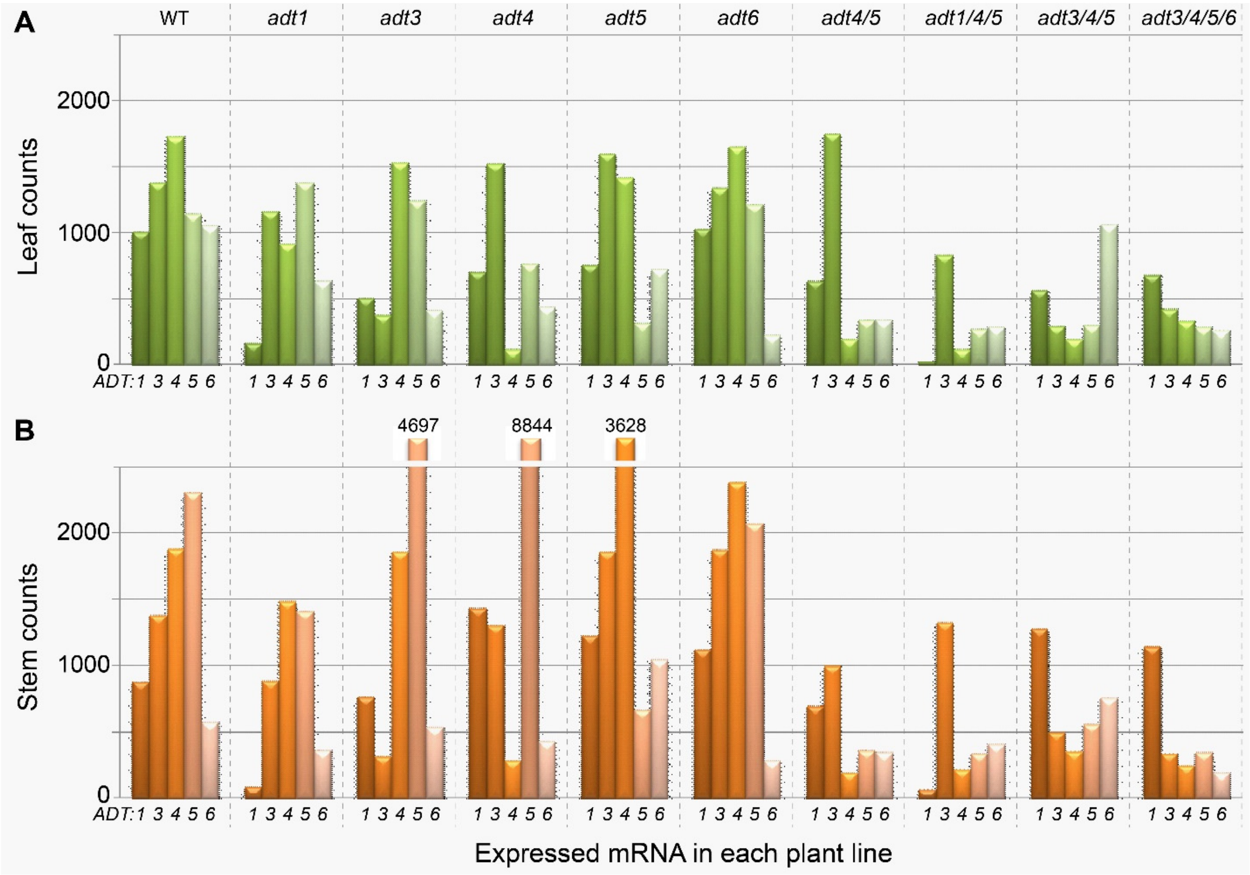

Supplementary Figure 4. RNA-Seq counts detected for each *ADT* gene transcript from each *ADT* KO mutant line or wild-type (WT) sample in **(A)** leaves or **(B)** stems.

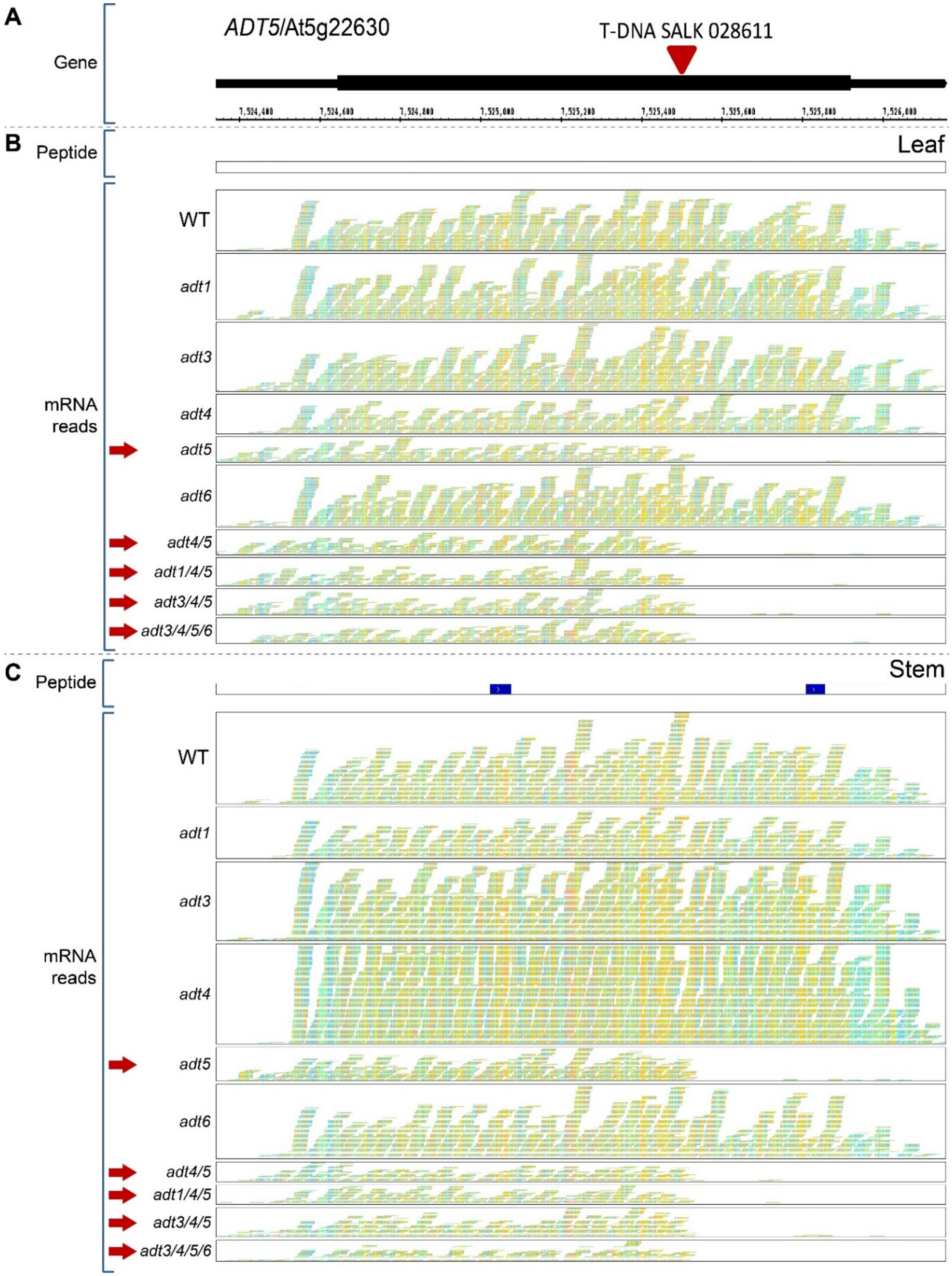

Supplementary Figure 5. Gene-transcript-peptide visualization in wild type (WT) and *ADT* KO mutant lines. **(A)** *ADT5* gene alongside T-DNA insertion placement. **(B)** Peptides and reads identified from the proteomics and transcriptomics data in leaf tissue. **(C)** Peptides and reads identified from the proteomics and transcriptomics data in stem tissue. Red arrows show which transcriptome samples contained the T-DNA insertion (depicted by a red triangle in **A**).

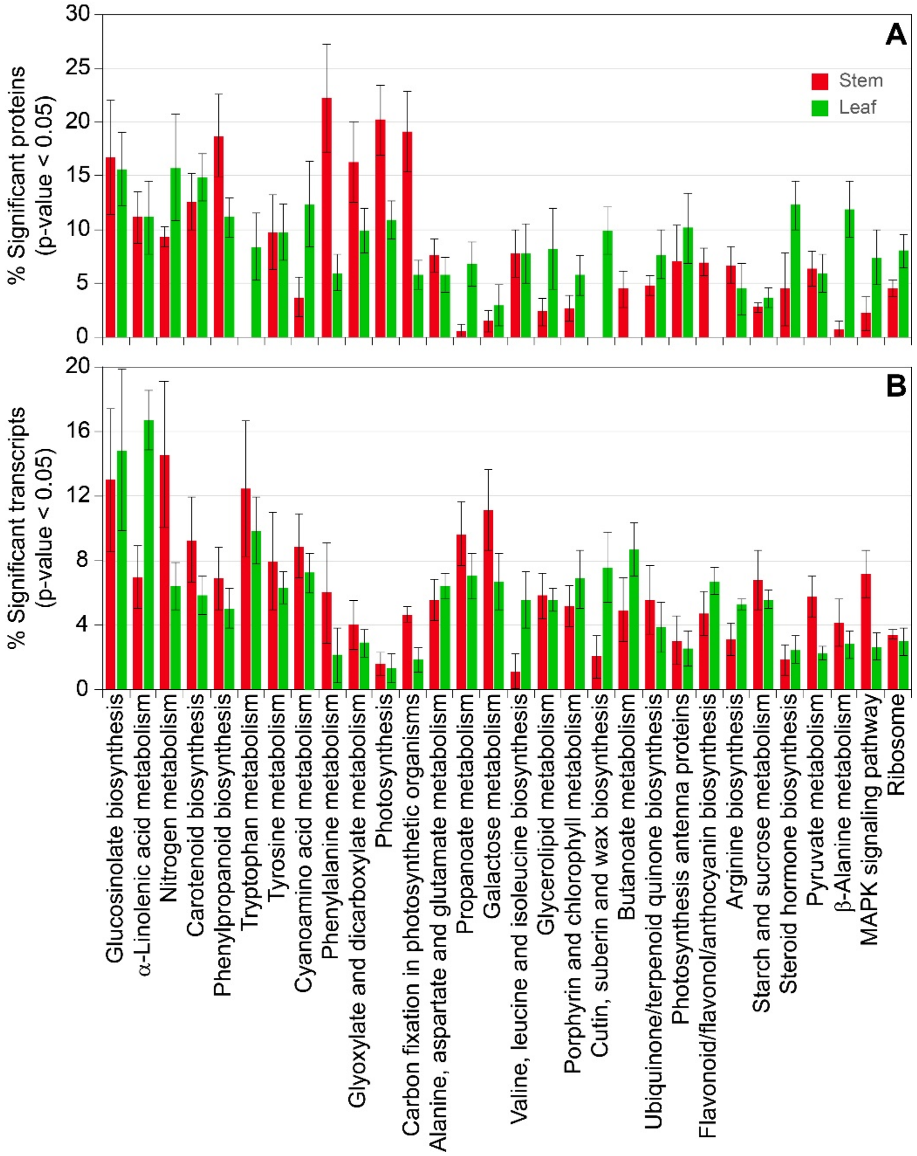

Supplementary Figure 6. KEGG functional categories which contained the most significant changes in *ADT* KO mutants. Average proportion of KEGG orthologs significantly changed (p-value <0.05) in each KEGG functional category for $\log_2$ **(A)** protein and **(B)** transcript ratio pairs (*ADT* KO mutant/WT) in 4-week old *Arabidopsis* stems and leaves. Functional categories were ranked from average highest value (leftmost) to lowest value (rightmost) for all protein and transcript data. Only the 30 most highly changed functional categories are displayed. Functional categories were only considered if they contained ten or more KEGG orthologs (i.e., isoenzyme families). Error bars represent standard error.

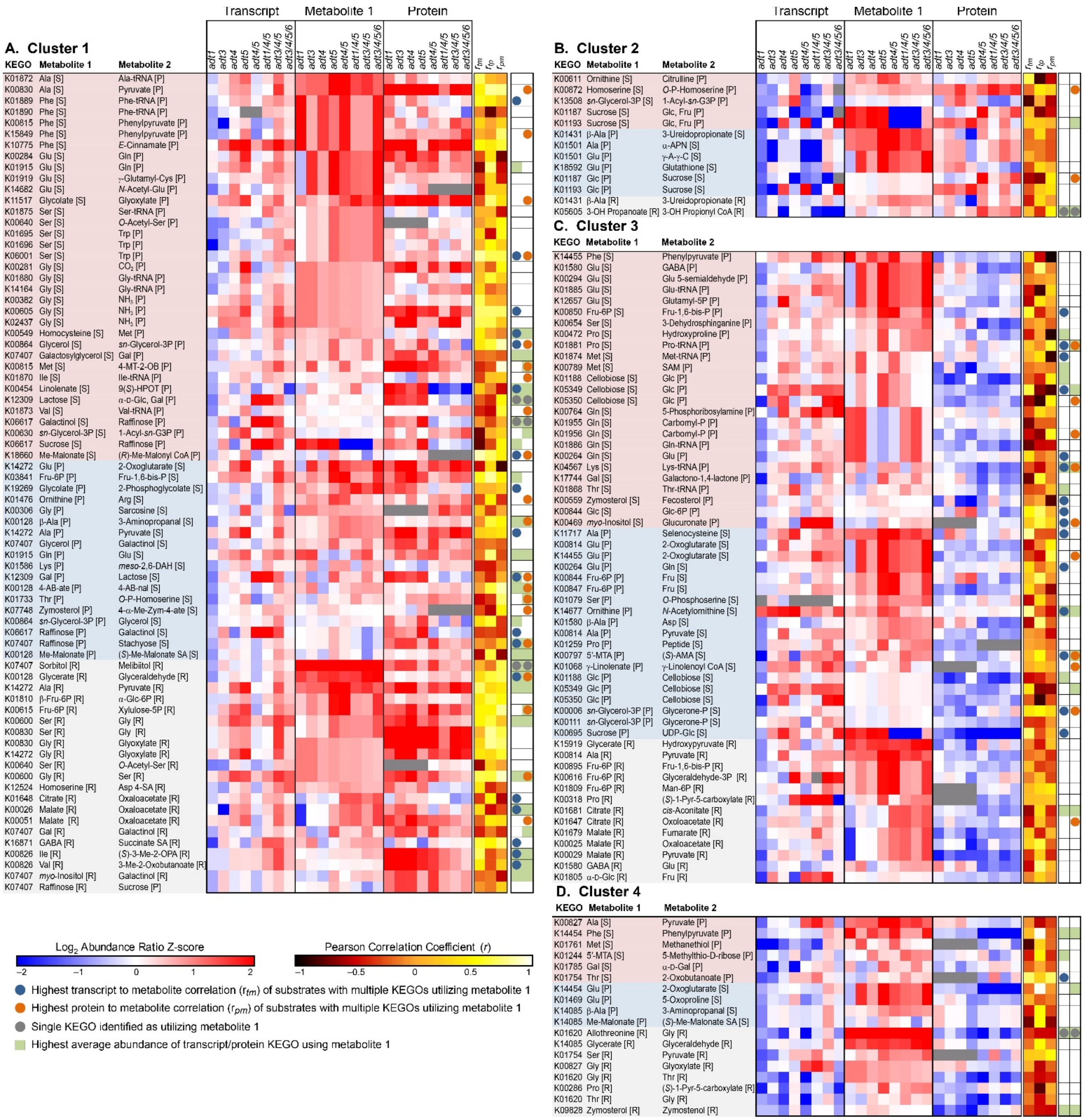

Supplementary Figure 7. Stem transcript-metabolite-protein heatmap displaying z-score comparisons of the log2 ratio pairs (*ADT* KO mutant/wild type, WT) of identified metabolites found to have a corresponding KEGG Ortholog Gene Family (KEGO) member which utilizes that specific metabolite as a substrate, and which were detected in both transcript and proteomics data. Each KEGO is displayed with substrate and product metabolite formed by that KEGO enzyme family. Data is further sorted into clusters that show: **(A) Cluster 1** – Transcripts, metabolites and proteins which all on average increased or decreased in the mutant KO lines together compared to WT. **(B) Cluster 2** – Transcripts decreased, while metabolites and proteins in the *ADT* KO lines, on average, increased in abundance. **(C) Cluster 3** – Transcripts and metabolites on average in the *ADT* KO lines increased, and proteins decreased in

abundance compared to WT. **(D) Cluster 4** – Transcripts and proteins decreased, and metabolites on average increased in the *ADT* KO mutants compared to WT.

In each cluster, entries are further grouped by whether or not Metabolite 1 is a substrate or product in a unidirectional reaction or if it can be utilized in a reversible reaction. Reactions are then ordered from highest average metabolite z-score to lowest metabolite z-score. Red represents metabolites higher in abundance in the *ADT* KO mutant compared to WT, blue represents metabolites higher in abundance in WT compared to the *ADT* KO mutant, white represents metabolites unchanged in abundance between WT and the *ADT* KO mutant, and grey represents constituents not detected. Green squares indicate the highest average KEGO value associated with each detected metabolite. Grey circles represent KEGO reactions where there was only a single known reaction for that given substrate-product reaction. Blue circles represent $\log_2$ transcript data that is most highly correlated to $\log_2$ metabolite data across *ADT* KO mutants, i.e., if ratio abundances between transcripts and metabolites both showed profile increases across single, double, triple and quadruple *ADT* KO mutants, those would have a positive correlation regardless if the z-score values themselves were negative or positive. Orange circles represent $\log_2$ protein data that are most highly correlated to $\log_2$ metabolite data across *ADT* KO mutants, i.e., if ratio abundances between proteins and metabolites both showed profile increases across single, double, triple and quadruple *ADT* KO mutants, those would have a positive correlation regardless if the z-score values themselves were negative or positive.

Abbreviations: r_{tm} = Pearson's Correlation between transcript and metabolite profiles. r_{pm} = Pearson's Correlation between protein and metabolite profiles. r_{tp} = Pearson's Correlation between transcript and protein profiles. 1-Acyl-*sn*-G3P = 1-Acyl-*sn*-glycerol 3-phosphate, (*S*)-1-Pyr-5-carboxylate = (*S*)-1-Pyrroline-5-carboxylate, 4-α-Me-Zym-4-ate = 4-α-Methylzymosterol-4-carboxylate, (*S*)-3-Me-2-OPA = (*S*)-3-Methyl-2-oxopentanoic acid, 4-MT-2-OB = 4-Methylthio-2-oxobutanoate, 5′-MTA = 5′-Methylthioadenosine, 9(*S*)-HPOT = 9(*S*)-Hydroperoxy-10*E*,12*Z*,15*Z*-octadecatrienoic acid, (*S*)-AMA = (*S*)-Adenosylmethioninamine, γ-A-γ-C = γ-Amino-γ-cyanobutanoate, α-APN = α-Aminopropiononitrile, GABA = 4-Aminobutanoate, Asp 4-SA = Aspartate 4-semialdehyde, *meso*-2,6-DAH = *meso*-2,6-Diaminoheptanedioate, (*S*)-Me-Malonate SA = (S)-Methylmalonate semialdehyde, 4-AB-ate = 4-Acetamidobutanoate, 4-AB-nal = 4-Acetamidobutanal, Succinate SA = Succinate semialdehyde.

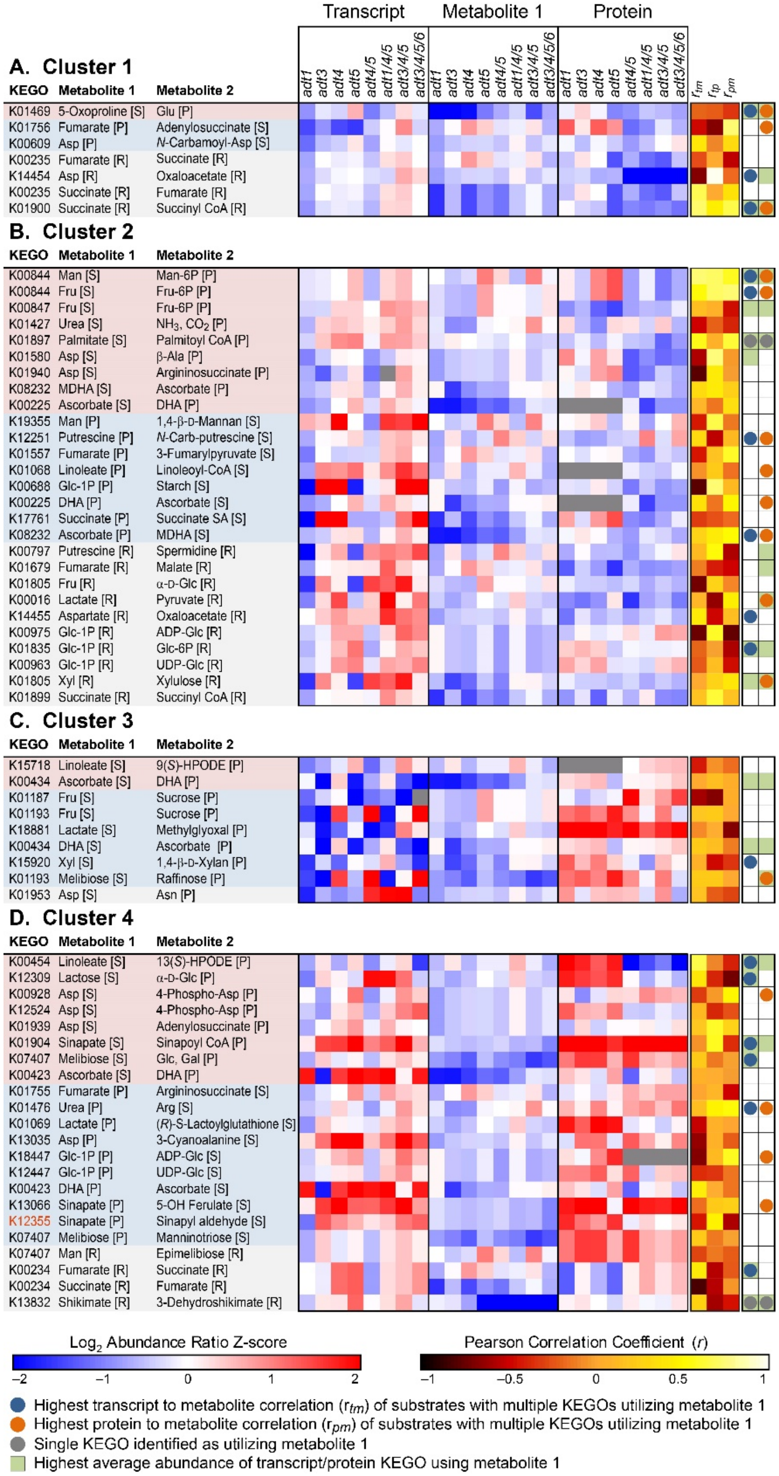

Supplementary Figure 8. Stem transcript-metabolite-protein heatmap displaying z-score comparisons of the $\log_2$ ratio pairs (*ADT* KO mutant/wild type, WT) of identified metabolites found to have a corresponding KEGG Ortholog Gene Family (KEGO) member which utilizes that specific metabolite as a substrate, and which were detected in both transcript and proteomics data. Each KEGO is displayed with substrate and product metabolite formed by that KEGO enzyme family. Data is further sorted into clusters that show: **(A) Cluster 1** – Transcripts, metabolites and proteins which all on average increased or decreased in the mutant KO lines together compared to WT. **(B) Cluster 2** – Transcripts decreased,

while metabolites and proteins in the *ADT* KO lines, on average, increased in abundance. **(C) Cluster 3** – Transcripts and metabolites on average in the *ADT* KO lines increased, and proteins decreased in abundance compared to WT. **(D) Cluster 4** – Transcripts and proteins decreased, and metabolites on average increased in the *ADT* KO mutants compared to WT. In each cluster, entries are further grouped by whether or not Metabolite 1 is a substrate or product in a unidirectional reaction or if it can be utilized in a reversible reaction. Reactions are then ordered from highest average metabolite z-score to lowest metabolite z-score. Red represents metabolites higher in abundance in the *ADT* KO mutant compared to WT, blue represents metabolites higher in abundance in WT compared to the *ADT* KO mutant, white represents metabolites unchanged in abundance between WT and the *ADT* KO mutant, and grey represents constituents not detected. Green squares indicate the highest average KEGO value associated with each detected metabolite. Grey circles represent KEGO reactions where there was only a single known reaction for that given substrate-product reaction. Blue circles represent $\log_2$ transcript data that is most highly correlated to $\log_2$ metabolite data across *ADT* KO mutants, i.e., if ratio abundances between transcripts and metabolites both showed profile increases across single, double, triple and quadruple *ADT* KO mutants, those would have a positive correlation regardless if the z-score values themselves were negative or positive. Orange circles represent $\log_2$ protein data that are most highly correlated to $\log_2$ metabolite data across *ADT* KO mutants, i.e., if ratio abundances between proteins and metabolites both showed profile increases across single, double, triple and quadruple *ADT* KO mutants, those would have a positive correlation regardless if the z-score values themselves were negative or positive.

Abbreviations: r_{tm} = Pearson's Correlation between transcript and metabolite profiles. r_{pm} = Pearson's Correlation between protein and metabolite profiles. r_{tp} = Pearson's Correlation between transcript and protein profiles. 1-Acyl-*sn*-G3P = 1-Acyl-*sn*-glycerol 3-phosphate, 9(*S*)-HPODE = 9(*S*)-Hydroperoxy octadecadienoic acid, 13(*S*)-HPODE = 13(*S*)-Hydroperoxy-(9*Z*,11*E*)-octadecadienoic acid, *N*-Carb-putrescine = *N*-Carbamoylputrescine, DHA = Dehydroascorbate, MDHA = Monodehydroascorbate, Succinate SA = Succinate semialdehyde.

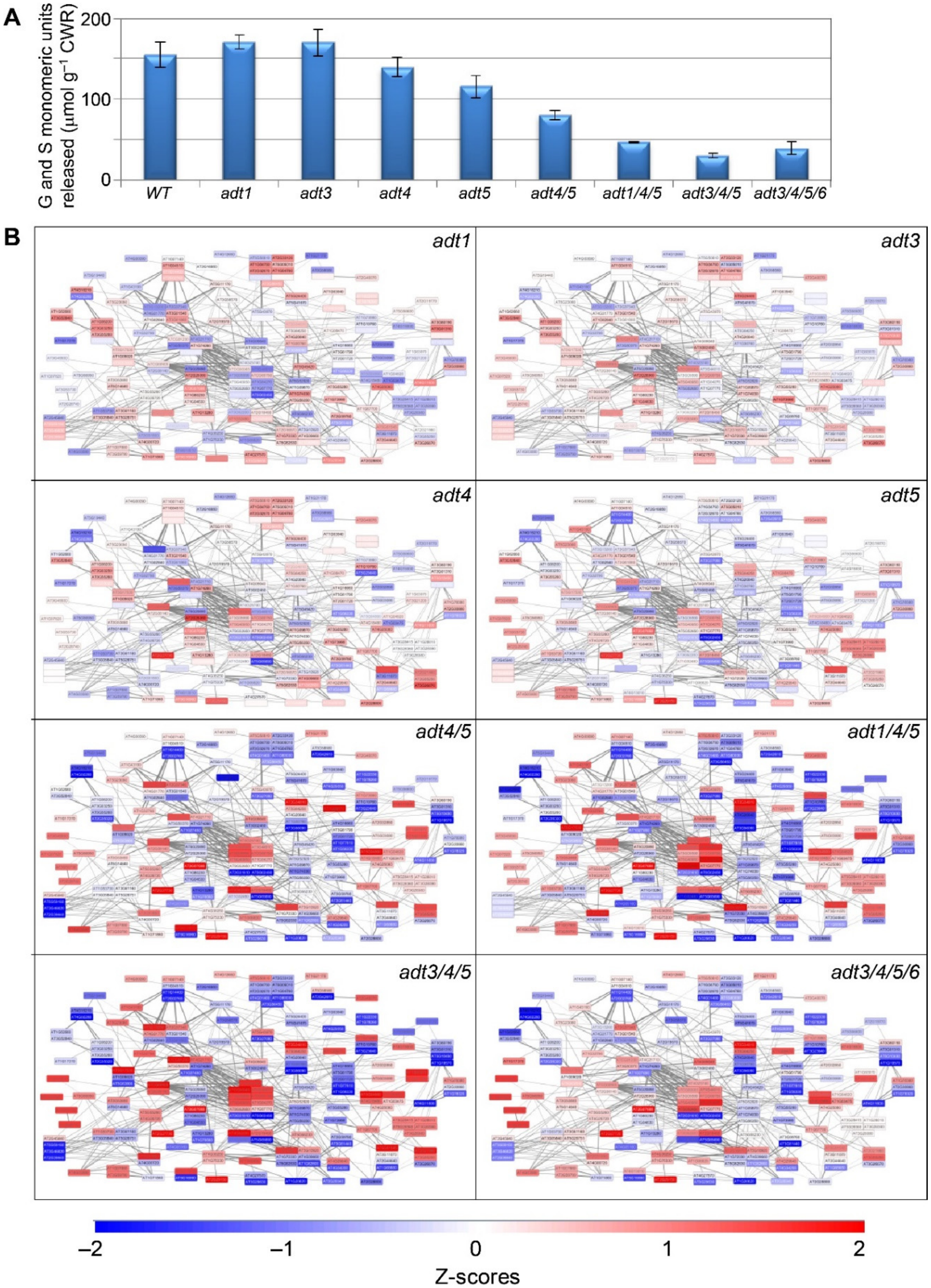

Supplementary Figure 9. (A) Measured guaiacyl (G) and syringyl (S) lignin monomer levels obtained from thioacidolysis of lignified tissues as reported in prior work (Corea et al., 2012). **(B)** Network analysis of relative protein abundances highly correlated as determined by Spearman Rank Correlations (rho >0.85) to monomeric G + S lignin-derived moieties measured in 4 week old stem tissues of *adt1,*

adt3, adt4, adt5, adt4/5, adt1/4/5, adt3/4/5, and adt3/4/5/6. Nodes are represented by rectangles colored by the z-score of the log_2 ratios (*ADT* KO mutant/wild type, WT), where bright yellow represents proteins higher in abundance in the *ADT* KO mutant compared to WT, bright blue represents proteins higher in abundance in WT compared to the *ADT* KO mutant, and black represents proteins unchanged in abundance between WT and the *ADT* KO mutant. This figure is meant to illustrate overall z-score trends observed in *ADT* KO mutants as lignin levels decrease. Like the levels of cleavable lignin G and S monomers, the most extreme changes are observed in stems with multiple *ADTs* knocked out. Refer to Figure 5A to decipher specific protein information.

References

Corea, O.R.A., Ki, C., Cardenas, C.L., Kim, S.J., Brewer, S.E., Patten, A.M., Davin, L.B., and Lewis, N.G. (2012). Arogenate dehydratase isoenzymes profoundly and differentially modulate carbon flux into lignins. *J. Biol. Chem.* 287, 11446–11459. doi: 10.1074/jbc.M111.322164

Kind, T., Wohlgemuth, G., Lee, D.Y., Lu, Y., Palazoglu, M., Shahbaz, S., and Fiehn, O. (2009). FiehnLib: Mass spectral and retention index libraries for metabolomics based on quadrupole and time-of-flight gas chromatography/mass spectrometry. *Anal. Chem.* 81, 10038–10048. doi: 10.1021/ac9019522
